# AmOctα2R: Functional Characterization of a Honeybee Octopamine Receptor Inhibiting Adenylyl Cyclase Activity

**DOI:** 10.3390/ijms21249334

**Published:** 2020-12-08

**Authors:** Wolfgang Blenau, Joana Alessandra Wilms, Sabine Balfanz, Arnd Baumann

**Affiliations:** 1 Institute of Biochemistry, Leipzig University, 04103 Leipzig, Germany; wolfgang.blenau@uni-leipzig.de; 2 Institute of Biological Information Processing, IBI-1, Research Center Jülich, 52428 Jülich, Germany; j.wilms@gmx.de (J.A.W.); s.balfanz@fz-juelich.de (S.B.)

**Keywords:** biogenic amines, cellular signaling, GPCR, honeybee, second messenger

## Abstract

The catecholamines norepinephrine and epinephrine are important regulators of vertebrate physiology. Insects such as honeybees do not synthesize these neuroactive substances. Instead, they use the phenolamines tyramine and octopamine for similar physiological functions. These biogenic amines activate specific members of the large protein family of G protein-coupled receptors (GPCRs). Based on molecular and pharmacological data, insect octopamine receptors were classified as either α- or β-adrenergic-like octopamine receptors. Currently, one α- and four β-receptors have been molecularly and pharmacologically characterized in the honeybee. Recently, an α_2_-adrenergic-like octopamine receptor was identified in *Drosophila melanogaster* (DmOctα2R). This receptor is activated by octopamine and other biogenic amines and causes a decrease in intracellular cAMP ([cAMP]_i_). Here, we show that the orthologous receptor of the honeybee (AmOctα2R), phylogenetically groups in a clade closely related to human α_2_-adrenergic receptors. When heterologously expressed in an eukaryotic cell line, AmOctα2R causes a decrease in [cAMP]_i_. The receptor displays a pronounced preference for octopamine over tyramine. In contrast to DmOctα2R, the honeybee receptor is not activated by serotonin. Its activity can be blocked efficiently by 5-carboxamidotryptamine and phentolamine. The functional characterization of AmOctα2R now adds a sixth member to this subfamily of monoaminergic receptors in the honeybee and is an important step towards understanding the actions of octopamine in honeybee behavior and physiology.

## 1. Introduction

The phenolamines tyramine and octopamine act as neurotransmitters, neuromodulators, and/or neurohormones in insects as well as other protostomes and play a significant role in the regulation of physiology and behavior of these animals (for recent reviews, see [1,2,3,4,5,6]). During the last decades, the honeybee (*Apis mellifera* (*A. mellifera*)) has become established as an important model organism for investigating the roles of biogenic amines on behavioral plasticity [7,8,9,10,11,12] and social behavior [13,14,15]. Physiologically, octopamine and tyramine are often considered to act similarly in the honeybee [16,17,18]. However, there is growing evidence for distinct effects of these two closely related amines on behavior in the bee [13,19,20] and the vinegar fly *Drosophila melanogaster* (*D. melanogaster*) [21,22,23,24,25,26]. Whether and how these effects can be traced back to the repertoire and the signaling capabilities of individual receptors is a challenging question.

Like other biogenic amines, tyramine and octopamine exert their actions by binding to the members of the superfamily of G protein-coupled receptors (GPCRs). For each phenolamine, there are multiple receptor subtypes that couple to various intracellular signaling pathways in a receptor subtype specific manner. In the honeybee, two tyramine receptors have been examined functionally so far. The AmTAR1 receptor (previously named AmTYR1) inhibits adenylyl cyclase activity and thus leads to a reduction in [cAMP]_i_ [27,28,29]. More recently, a second tyramine receptor, AmTAR2, has been characterized that specifically induces cAMP production upon activation [30]. The family of octopamine receptors in the honeybee is more complex [31,32]. At the cellular level, these receptors evoke Ca^2+^ release from intracellular stores (AmOctαR, previously named AmOA1 [31]) or activate adenylyl cyclases, thereby increasing [cAMP]_i_ (AmOctβR1–4 [32]). Recently, a novel octopamine receptor subtype was characterized in the rice stem borer, *Chilo suppressalis* (*C. suppressalis*; CsOctα2R = CsOA3 [33]) and *D. melanogaster* (DmOctα2R; CG18208 [34]). Interestingly, the activation of DmOctα2R resulted in inhibition of forskolin-stimulated cAMP synthesis [34]. Thus, a signaling pathway is activated that was formerly not known to be used by octopamine. In addition, DmOctα2R displays an unusual pharmacological profile and is also activated by tyramine and the indoleamine serotonin in a dose-dependent manner [34].

A gene potentially encoding an α_2_-adrenergic-like octopamine receptor was also identified in the honeybee genome [33,34,35]. The aim of the current study was to molecularly and pharmacologically characterize this AmOctα2R receptor. Therefore, upon cloning the complete coding sequence from honeybee brain cDNA, we constitutively expressed AmOctα2R in a cell line and examined its coupling to intracellular second messengers and its pharmacological properties. Intriguingly, receptor activation with octopamine led to a decrease in [cAMP]_i_. We showed that AmOctα2R had a clear preference for octopamine over tyramine (~30-fold difference in half-maximal reduction of cAMP levels (EC_50_)). In contrast to DmOctα2R, however, AmOctα2R was not activated by serotonin. We concluded that in vivo effects of octopamine on second messenger signaling depended on the tissue- and cell-type-specific expression patterns of the various receptor subtypes and, additionally, on potential cross-activation of tyramine receptors.

## 2. Results

### 2.1. Molecular and Structural Properties of AmOctα2R

The amino acid sequence of a potential α_2_-adrenergic-like octopamine receptor from the honeybee has been annotated in previous studies [33,34,35]. Here, we have cloned the complete cDNA-encoding AmOctα2R by PCR on single-stranded cDNA synthesized on mRNA of adult worker bee brains. The cDNA contained an open reading frame (ORF) of 2223 bp. The corresponding gene was located on chromosome LG15 (see NCBI: NC_007084.3) and consisted of three exons (Supplementary Figure S1 and Table S1).

**Figure 1:**
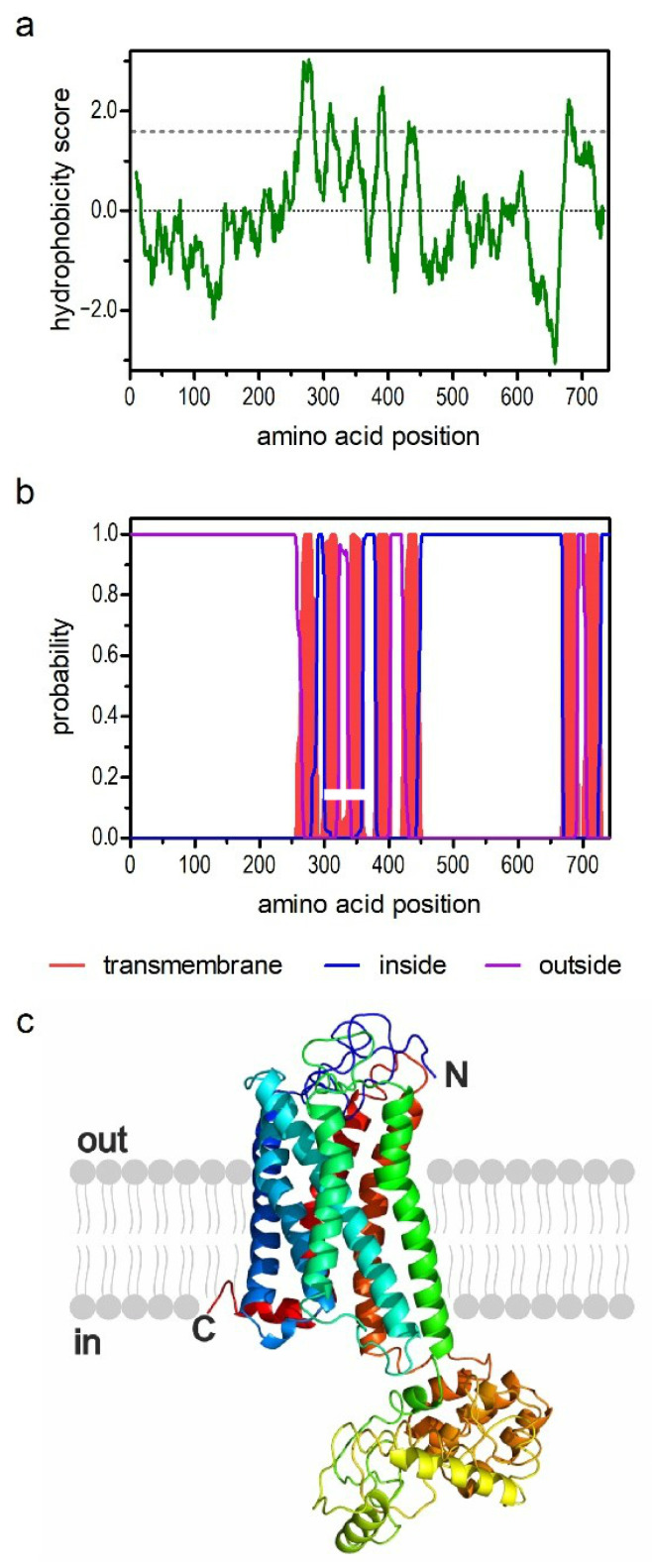
Structural characteristics of the amino acid sequence deduced for AmOctα2R. (**a**) Hydrophobicity profile of AmOctα2R. The profile was calculated according to the algorithm of Kyte and Doolittle [36] using a window size of 19 amino acids. Peaks with scores greater than 1.6 (dashed line) indicate possible transmembrane (TM) regions; (**b**) prediction of TM domains with TMHMM server v. 2.0 [37]. Putative TM domains are indicated in red. Extracellular regions are shown with a purple line, and intracellular regions are shown with a blue line; (**c**) color-coded (rainbow) three-dimensional (3D) model of the receptor as predicted by Phyre2 [38]. The extracellular N-terminus (N) and the intracellular C-terminus (C) are labeled. Note that the first 216 amino acid residues of AmOctα2R were omitted in this simulation.

The deduced amino acid sequence consisted of 741 residues with a calculated molecular weight of 80.7 kDa. The hydrophobicity profile according to Kyte and Dolittle [36] and the prediction of transmembrane (TM) helices using TMHMM Server v.2.0 [37] suggested seven TM domains (Figure 1a,b), which is a characteristic feature of GPCRs. The TM segments were flanked by an extracellular N-terminus of 263 residues and a short intracellular C-terminus of 14 residues. We submitted the AmOctα2 sequence to Phyre2 [38] and obtained a three-dimensional model of the receptor. In this model, the N-terminus was almost unstructured and loosely attached to the TM domains eventually crossing the membrane as an eighth TM segment. We, therefore, omitted the first 217 residues of the primary structure and recalculated the model from residue 218 to 741. This was revealed in the typical membrane arrangement of a GPCR (Figure 1c).

The sequence of AmOctα2R contained several putative sites for posttranslational modification (Supplementary Figure S2). Four potential N-glycosylation sites (N-X-(S/T)) were present in the extracellular N-terminus: N_27_MT, N_164_NT, N_238_GS, and N_243_ET. Conserved cysteine residues (C_336_ and C_414_) in the first and second extracellular loops might form a disulfide bridge as found in other biogenic amine receptors [39]. Five consensus sites for phosphorylation by protein kinase C and one consensus site for phosphorylation by protein kinase A were found in the cytoplasmic domains of the receptor protein (Supplementary Figure S2).

In addition to these sites, several cognate sequence motifs of GPCRs were identified in the primary structure of AmOctα2R. The D_360_RY motif (D^3.49^R^3.50^Y^3.51^; labeled according to [40] was located at the cytoplasmic end of TM3. In TM7, the residues N_720_PFIY (N^7.49^P^7.50^F^7.51^I^7.52^Y^7.53^) constituted part of the hydrophobic interaction site with the phenyl moiety of the biogenic amine. Furthermore, residues that most likely bound to the ligand (e.g., D_343_ (D^3.32^) and S_426/430_ (S^5.42/5.46^)) were highly conserved within the family of biogenic amine receptors [41].

**Figure 2:**
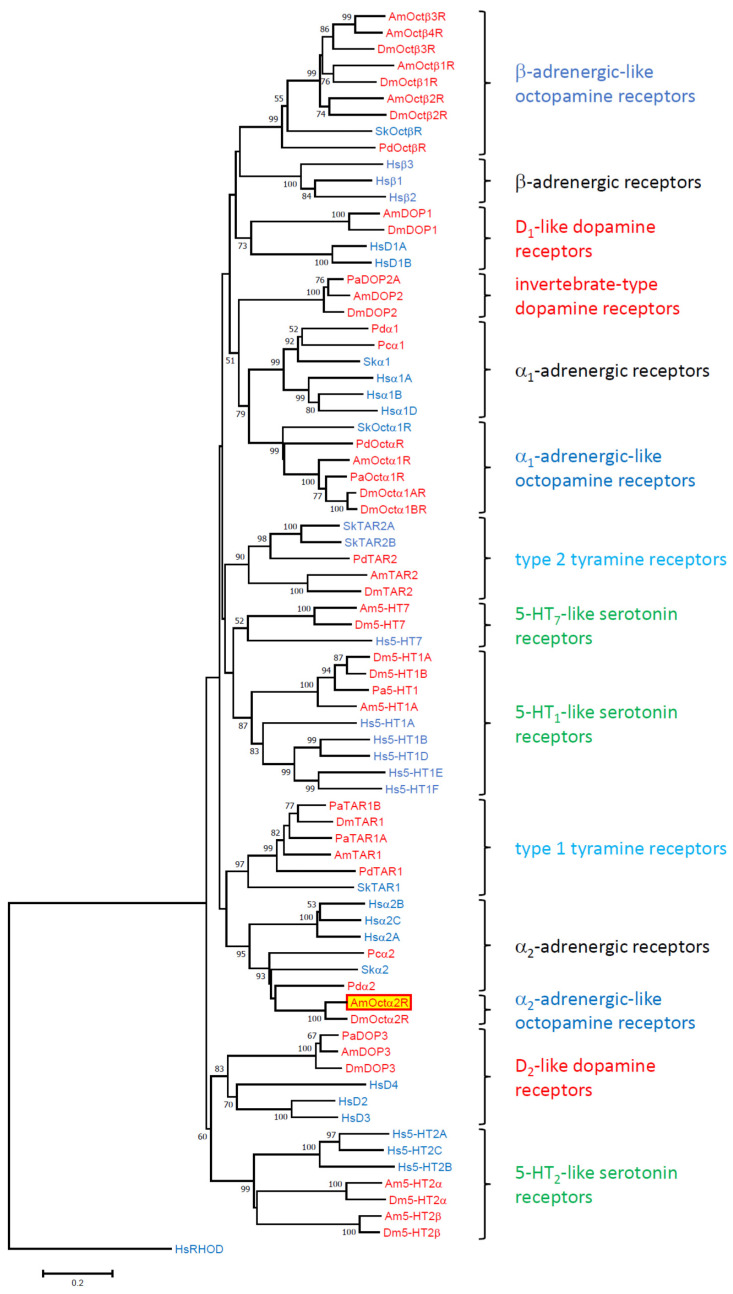
Phylogenetic relationships of monoaminergic receptors. Alignments were performed using Clustal W [46] by using the core amino-acid sequences of TM 1–4, TM 5, TM 6, and TM 7. The evolutionary history was inferred using the neighbor-joining method. The percentage of replicate trees, in which the associated taxa clustered together in the bootstrap test (10,000 replicates), are shown next to the branches. The tree was drawn to scale, with branch lengths in the same units as those of the evolutionary distances used to infer the phylogenetic tree. The evolutionary distances were computed using the Poisson correction method and are in the units of the number of amino acid substitutions per site. The analysis involved 76 amino acid sequences. Human rhodopsin (HsRHOD) was used to root the tree. Receptor subclasses are given on the right. The abbreviations of species are shown in alphabetical order: Am, *Apis mellifera*; Dm, *Drosophila melanogaster*; Hs, *Homo sapiens*; Pa, *Periplaneta americana*; Pc, *Priapulus caudatus*; Pd, *Platynereis dumerilii*; Sk, *Saccoglossus kowalevskii*. Protostomian species names are highlighted in red, whereas deuterostomian species names are given in blue. The accession numbers and annotations of all sequences used in the phylogenetic analysis can be found in Supplementary Table S2.

The phylogenetic relationship of the AmOctα2R receptor was examined using MEGA7 software (Figure 2). Not all receptors binding to a certain biogenic amine were composed of uniform clusters, but the appropriate receptor subgroups did. AmOctα2R assembled in a clade that contained an α_2_-adrenergic-like octopamine receptor from *D. melanogaster* [34] and α_2_ adrenergic receptors from *Platynereis dumerilii* (Pdα2 [42]), *Saccoglossus kowalevskii* (Skα2 [42]), and *Priapulus caudatus* (Pcα2 [42]). This clade was closely related to human α_2_-adrenergic receptors. In contrast, α_1_-adrenergic-like octopamine receptors including AmOctα1R [31] were clearly set apart and formed a sister group with α_1_-adrenergic receptors (Figure 2). Both α_1_-adrenergic-like octopamine receptors and α_1_-adrenergic receptors were also closely related to the invertebrate-type dopamine receptors from *A. mellifera* (AmDOP2 [43]), *Periplaneta americana* (*P. americana*; PaDOP2 [44]), and *D. melanogaster* (DmDOP2 [45]).

The complete primary structures of the AmOctα1R [31] and AmOctα2R receptors were only 22.1% identical and 33.5% similar. Notably, AmOctα2R was more closely related to α_2_-adrenergic-like octopamine receptors from the striped rice stemborer *C. suppressalis* (47.8%/55.1%), the red flour beetle *Tribolium castaneum* (46.3%/53.3%), and *D. melanogaster* (43.3%/50.8%).

### 2.2. Expression of AmOctα2R-HA in flpTM Cells

**Figure 3:**
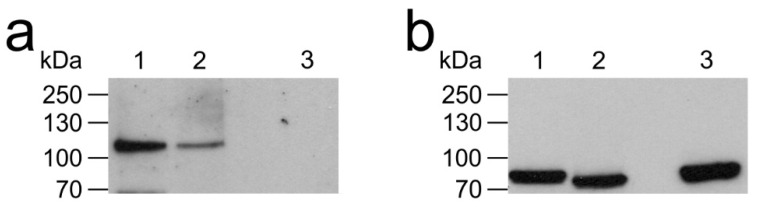
Expression of AmOctα2R-hemagglutinin A (HA) in flpTM cells. (**a**) Western blot of membrane proteins (30 µg) from flpTM cells expressing AmOctα2R-HA receptors were not treated (lane 1) or treated with PNGaseF (lane 2). As a control, 30 µg of membrane proteins from nontransfected flpTM cells (lane 3) were separated by sodium dodecyl sulfate-polyacrylamide gel electrophoresis (SDS-PAGE) and blotted to a polyvinylidene difluoride (PVDF) membrane. The blot was probed with a rat anti-(hemagglutinin A) HA antibody. (**b**) The same blot as shown in (**a**) was subsequently probed with an antibody directed against the C-terminus of the cyclic nucleotide-gated (CNG) channel. The sizes of marker proteins in kDa are given on the left margin.

To unravel the intracellular signaling pathway activated by AmOctα2R-HA and determine its pharmacological properties, flpTM cells were stably transfected with the expression construct. Independent cell lines were obtained and examined by immuno-fluorescence staining for homogeneity (Supplementary Figure S3). Additionally, AmOctα2R-HA was examined by Western blotting (Figure 3). The anti-(hemagglutinin A) HA antibody labeled a band of ~117 kDa (Figure 3a, lanes 1 and 2), which was absent in flpTM cells (Figure 3a, lane 3). Thus, the apparent molecular weight of the receptor was significantly greater than the calculated molecular weight of AmOctα2R-HA (81.8 kDa). Whether this difference was due to glycosylation was assessed by treating samples with and without PNGaseF separately. The mobility of the protein was not altered by PNGaseF treatment (Figure 3a, lanes 1 and 2), suggesting that AmOctα2R-HA was not glycosylated in these cells. As an internal control, the blot was developed with an antibody directed against the cyclic nucleotide-gated (CNG) channel (Figure 3b). In this case, the treatment with PNGaseF resulted in a reduction of the apparent molecular weight of the channel protein (Figure 3b, lane 2). Therefore, the difference between the apparent and calculated molecular weights of AmOctα2R-HA is possibly due to receptor dimerization and other post-translational modifications, e.g., phosphorylation or unusual electrophoretic mobility under denaturing conditions.

### 2.3. Ligand Specificity of the AmOctα2R-HA Receptor

The α_2_-adrenergic octopamine receptors from *C. suppressalis* [33] and *D. melanogaster* [34] have been shown to attenuate [cAMP]_i_ upon activation. We examined whether AmOctα2R-HA might also couple to G_i_-type G proteins, thereby causing inhibition of cell-endogenous adenylyl cyclases. To examine AmOctα2R-HA’s coupling properties, cells were treated with a water-soluble forskolin analog, NKH 477, which stimulated membrane-bound adenylyl cyclases. NKH 477 led to cAMP production in both nontransfected and AmOctα2R-HA-expressing flpTM cells. Next, we assessed the effects of the biogenic amines octopamine, tyramine, dopamine, and serotonin (10^−6^ M each) on NKH 477-stimulated cAMP production. The application of octopamine and tyramine led to a decrease in the Ca^2+^-dependent fluorescence signal, whereas the other amines had no effect on such signals. Cells that did not express the receptor (flpTM) showed no Ca^2+^-dependent responses after the application of biogenic amines.

**Figure 4:**
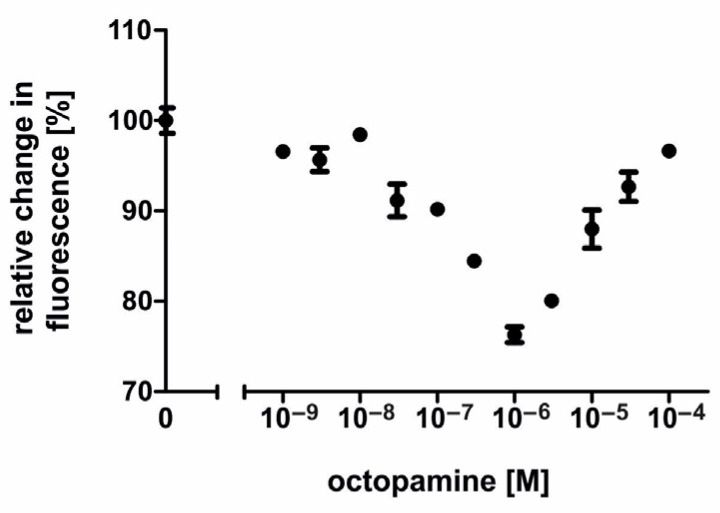
Concentration-dependent effects of octopamine on intracellular cAMP in AmOctα2R-HA-expressing flpTM cells. Relative fluorescence (corresponding to the amount of cAMP) is given as the percentage of the value obtained with 10 µM NKH 477 (=100%), a water-soluble forskolin analog. All measurements were performed in the presence of 100 µM isobutylmethylxanthine (IBMX). In the range from 10^−9^ M to 10^−6^ M, the octopamine activation of AmOctα2R-HA led to a concentration-dependent decrease in the fluorescence signal. Conversely, an increase in the fluorescence signal was observed with octopamine concentrations of 3 × 10^−6^ M and higher. Data points represent the mean ± SD of four-fold determinations.

**Figure 5:**
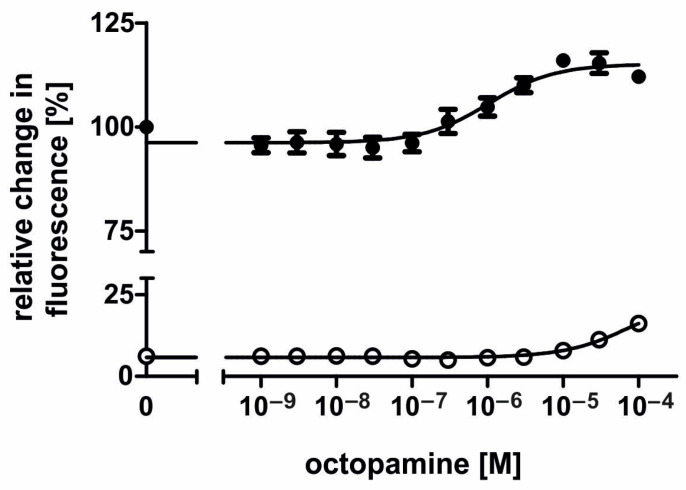
Concentration-dependent effects of octopamine on relative fluorescence in nontransfected (control) flpTM cells. The concentration–response curves for octopamine were established in the absence (open circles) or presence (filled circles) of 10 µM NKH 477. Relative fluorescence is given as the percentage of the value obtained with 10 μM NKH 477 (=100%). All measurements were performed in the presence of 100 μM IBMX. In both conditions, higher octopamine concentrations led to an increase in fluorescence. Data points represent the mean ± SD of four values.

To further investigate AmOctα2R-HA’s properties, the concentration–response curves for octopamine and tyramine were established. Octopamine was applied in concentrations ranging from 10^−9^ M to 10^−4^ M. Unexpectedly the concentration–response curve was U-shaped (Figure 4). A decrease in fluorescence was observed with octopamine concentrations ranging from 10^−9^ M to 10^−6^ M. Considering octopamine concentrations from 10^−9^ M to 3 × 10^−6^ M, EC_50_ was observed with 1.17 × 10^−7^ M octopamine (logEC_50_ ± SD = −6.932 ± 0.1395; for the mean values of all experiments, see Table 1). The maximal reduction of cAMP synthesis was ~25% at 10^−6^ M octopamine. Octopamine concentrations higher than 10^−6^ M led to an increase in Ca^2+^-dependent fluorescence signals (Figure 4), suggesting that the parental flpTM cell line expresses receptors that could be activated by octopamine and cause either a cAMP response and/or direct Ca^2+^ responses [47]. To test this hypothesis, nontransfected flpTM cells were incubated with increasing octopamine concentrations (Figure 5). In the presence of NKH 477, octopamine concentrations of ≥ 3 × 10^−7^ M led to an increase in Fluo-4 fluorescence, which argued for the presence of such endogenous receptors. Although we did not address the molecular identity of these receptors, they most likely belong to the family of the adrenergic GPCRs that have been previously found in these cells [48].

**Figure 6:**
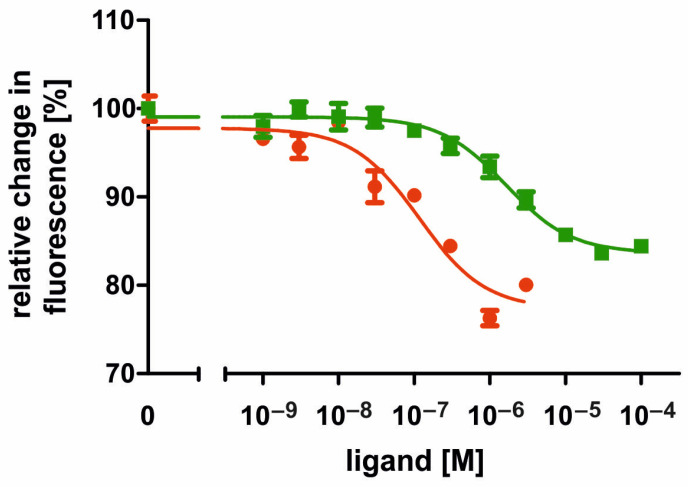
Concentration-response curves for agonists on intracellular cAMP level in AmOctα2R-HA-expressing flpTM cells. Relative fluorescence (corresponding to the amount of cAMP) is given as the percentage of the value obtained with 10 µM NKH 477 (=100%). All measurements were performed in the presence of 100 µM IBMX. Data points represent the mean ± SD of four values from a typical experiment.

The concentration–response curve for tyramine was sigmoid and saturated at a tyramine concentration of ≥3 × 10^−5^ M (~17% reduction; Figure 6). The ligand concentration leading to the half-maximal activation of AmOctα2R-HA (EC_50_) was 1.628 × 10^−6^ M tyramine (logEC_50_ ± SD = −5.788 ± 0.092; for the mean values of all experiments, see Table 1). In nontransfected flpTM cells, no change in the fluorescence signal was observed upon application of tyramine. Accordingly, all subsequent measurements with antagonists (see below) were first carried out against a tyramine background. In conclusion, the results indicated that AmOctα2R-HA has a clear (~30-fold) specificity for octopamine over tyramine and can be considered a functional α_2_-adrenergic-like octopamine receptor.

### 2.4. Pharmacological Properties of the AmOctα2R-HA Receptor

**Figure 7:**
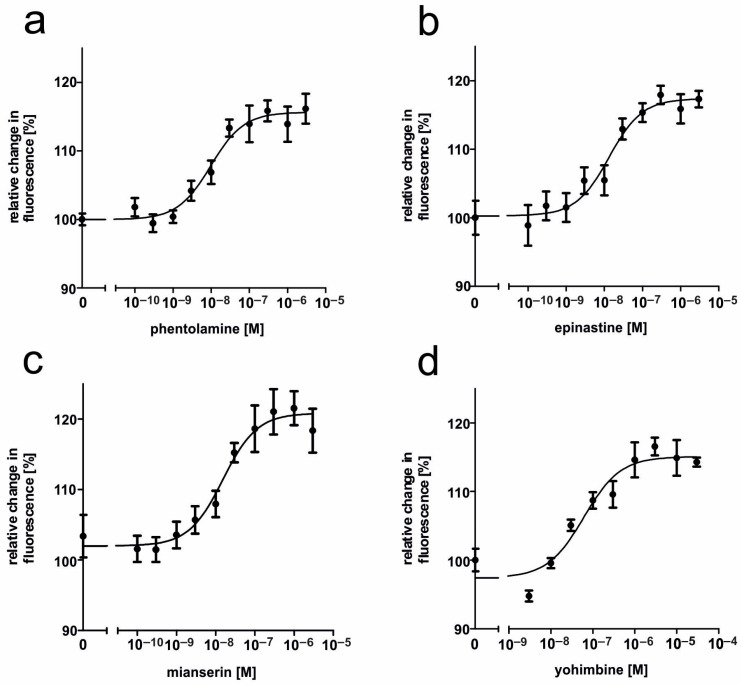
Effects of putative antagonists on tyramine-activated AmOctα2R-HA. The concentration series of the substances were applied in the presence of 10 µM NKH 477, 10 µM tyramine, and 100 µM IBMX. Ligands used were (**a**) phnetolamine, (**b**) epinastine, (**c**) mainserin, and (**d**) yohimbine. Data represent the mean ± SD of four values from a typical experiment. All determinations were independently repeated at least three times.

The ability of various potential antagonists for impairing AmOctα2R-HA activity was assessed in a similar way. Measurements were performed with increasing concentrations of antagonists AS-19, 5-carboxamidotryptamine (5-CT), 5-methoxytryptamine (5-MT), 8-Hydroxy-2-(dipropylamino)tetralin (8-OH-DPAT), epinastine, ketanserin, mianserin, phentolamine, and yohimbine on a nonsaturating tyramine background (10 µM). In NKH 477-treated and tyramine-stimulated AmOctα2R-HA expressing cells, the application of antagonists led to an increase in the fluorescence signal, because adenylyl cyclases were no longer inhibited by G_i_-proteins. In Figure 7, the antagonistic effects of phentolamine, epinastine, mianserin, and yohimbine are displayed. Ligand concentrations that led to the half-maximal inhibition of AmOctα2R-HA (IC_50_) were determined from the concentration–response curves and are summarized in Table 2. Effective antagonists of tyramine-stimulated AmOctα2R-HA were, for example, 5-CT and phentolamine with IC_50_ values of 4.16 × 10^−9^ M and 5.6 × 10^−9^ M. The order of antagonist potency of tyramine-stimulated AmOctα2R-HA was 5-CT ≥ phentolamine > epinastine > 5-MT > mianserin > yohimbine > ketanserin > 8-OH-DPAT (for mean values of IC_50_, see Table 2). AS-19 did not show any effect.

Substances, which showed antagonistic activity at AmOctα2R-HA against a tyramine background, were also tested against an octopamine background (3 × 10^−7^ M). The rank order of potency was similar to measurements performed with tyramine. The mean values for half-maximal inhibition (IC_50_ [M] and logIC_50_ ± SD) are summarized in Table S3.

## 3. Discussion

There is ongoing interest in precisely understanding the physiological and behavioral roles of octopaminergic signaling in insects [2,6,26,49,50,51]. An important step to meet this challenge is to determine the molecular and functional pharmacological properties of octopamine receptor subtypes. Here, we described the functional characterization of AmOctα2R, the sixth octopamine receptor subtype of the honeybee. The primary structure of AmOctα2R phylogenetically clustered with protostomian α_2_-adrenergic-like octopamine receptors. Activation of AmOctα2R by the phenolamines octopamine and tyramine led to a substantial decrease of NKH 477-induced cAMP synthesis. In contrast to DmOctα2R from *D. melanogaster* [34], we did not observe any changes in [cAMP]_i_ in response to the indoleamine serotonin.

### 3.1. Gene Structure, Structural Properties of the Protein, and Phylogenetic Classification

The coding region of the *AmOctα2R* gene was dispersed over approximately 13 kb of genomic DNA on the linkage group LG15 and was interrupted by two introns. The gene was located on the same chromosome, as the *AmOctα1R* gene of which the coding region was interrupted by eight introns (Table S1, Figure S1; [31,35]). Whereas the position of intron 1 was conserved between orthologous receptors of *A. mellifea* and *D. melanogaster*, this is not the case for intron 2 (Figure S1). Since amplification on brain cDNA resulted in only one distinct product, we found no evidence for alternative splicing of the *AmOctα2R* transcript, as has been described for transcripts of orthologous receptors of both *C. suppressalis* [33] and *D. melanogaster* [34].

Applying several in silico analyses confirmed that AmOctα2R is a member of the class A (rhodopsin-like) GPCR family. This assessment was supported by the presence of cognate amino acid residues and motifs within the TM segments in AmOctα2R, e.g., N_720_PFIY in TM7 or the D_360_RY motif at the C-terminal end of TM3.

Most class A (rhodopsin-like) GPCRs were activated by ligands docking to specific residues in the binding pocket of the receptor near the extracellular side. Functionally important amino acid residues present in α_2_-adrenergic-like octopamine receptors were well conserved in the AmOctα2R sequence. They were an aspartic acid residue (D_342_) in TM3 and two of three closely grouped serine residues found in TM5 (S_426,430_) (see Supplementary Figure S2). Octopamine appeared to bind via its amine group and its hydroxyl group to the aspartic acid and one of the serine residues of the receptor, respectively [52,53]. In addition, phenylalanine and/or tryptophan residues in TM6 and TM7 (see Supplementary Figure S2) might contribute to π–π interaction with delocalized electrons in octopamine or tyramine and stabilize the receptor ligand interaction.

The coupling of GPCRs to specific G proteins is brought about by amino-acid residues in close vicinity to the plasma membranes of the 2nd and 3rd intracellular loops and of the cytoplasmic C-terminus of the receptor [54,55,56]. Biogenic amine receptors that couple to G_i_ proteins and thereby inhibit adenylyl cyclase activity often possess short C termini [54]. This feature is conserved in AmOctα2R and in other α_2_-adrenergic-like octopamine receptors (Supplementary Figure S2; [33,34]). In addition, the receptors possess strikingly similar amino-acid sequences in the vicinity of TM5 and TM6 within their 3rd cytoplasmic loops, a region largely determining the specificity of receptor/G protein coupling [57].

Our phylogenetic analysis including all major insect biogenic amine GPCR families resulted in a well-resolved phylogram (Figure 2). Protostomian α_2_-adrenergic-like octopamine receptors seemed to be closely related to deuterostomian α_2_-adrenergic receptors, emphasizing the idea of “ligand-hopping” during evolution of aminergic GPCRs [35]. When new receptors evolved by gene duplication, they needed new ligands. Because of structural constraints, the only way to obtain “new” aminergic ligands was to repurpose already existing biogenic amines from other systems. The frequent ligand exchanges during evolution of aminergic GPCRs strongly contrasted the situation observed for neuropeptide and protein hormone GPCRs, where generally co-evolution between receptors and their ligands takes place [35,58,59].

The sister group of both α_2_-adrenergic-like octopamine and α_2_-adrenergic receptors constituted type 1 tyramine receptors which also couple to G_i_ proteins (Figure 2; [33,34]). A slightly different assignment of the α_2_-adrenergic-like octopamine receptor family has been described in an independent study. Here, the phylogenetic trees were calculated with RAxML using the CIPRES Science Gateway [42]. The α_2_-adrenergic-like octopamine receptors assembled at the basal branches of the dendrogram forming the sister group of all other tyramine-, octopamine-, and adrenergic receptors. The different results may originate from the strategies applied in creating the datasets used for calculating the phylograms. Based on our results, we suggest including all major receptor families to unravel the evolutionary relationship of biogenic amine receptors.

### 3.2. Posttranslational Modification of AmOctα2R

Posttranslational modifications in intracellular loops of AmOctα2R, like phosphorylation, may also affect the signaling properties of the protein. It has been recently shown that phosphorylation of a single residue in the third intracellular loop of an octopamine receptor from *D. melanogaster* (DmOctαR1B; [60,61] is sufficient to explain the receptor’s oscillatory Ca^2+^ signaling behavior [62]. Whether transient changes in the surface charge of AmOctα2R also lead to oscillatory phases of adenylyl cyclase inhibition remains to be addressed. Cysteine residues in the C-terminus of different biogenic amine receptors were found to undergo posttranslational palmitoylation [63]. This modification generates a fourth intracellular loop that also participates in receptor–G protein binding [59]. Since a cysteine is missing in the C-terminus of AmOctα2R, the fourth intracellular loop does not exist in this GPCR.

### 3.3. Pharmacological Properties of the AmOctα2R Protein

The AmOctα2R receptor was functionally expressed in flpTM cells. The coupling of AmOctα2R to intracellular signaling cascades was examined via cell-endogenous G proteins. AmOctα2R, like other α_2_-adrenergic-like octopamine receptors from insects [33,34] and mammalian α_2_-adrenergic receptors (for a review, see [64]), was negatively coupled to the enzyme adenylyl cyclase via G_i_ proteins and thus resulted in a decrease in [cAMP]_i_. With a mean EC_50_ of 58.7 nM, activation of AmOctα2R was much more sensitive to octopamine than to tyramine (mean EC_50_ = 1.85 µM; Table 1). These data agree well with those described for orthologous receptors [33,34]. Interestingly, besides cAMP signaling, the addition of octopamine, tyramine (and dopamine) to CsOctα2R-expressing HEK 293 cells also resulted in concentration-dependent increases in [Ca^2+^]_i._ This has not been found for DmOctα2R- [34] or AmOctα2R-expressing cells (this study). However, apart from obvious similarities in the pharmacological properties, there were also significant differences between DmOctα2R and AmOctα2R. Whereas DmOctα2R was activated by serotonin in a dose-dependent manner (EC_50_ = 1.04 µM; [34]), serotonin failed to activate AmOctα2R.

The inhibition of AmOctα2R-HA-mediated attenuation of [cAMP]_i_ in the cell line was examined with various synthetic antagonists. In addition to phentolamine (IC_50_ = 5.6 nM/8.21 nM) which is a nonselective α-adrenergic antagonist (for a review, see [65]), the action of tyramine and octopamine on AmOctα2R could also be blocked by 5-CT (IC_50_ = 4.16 nM/0.27 nM) with even slightly higher potency. The substance 5-CT is primarily known as an agonist at 5-HT_1A_, 5-HT_1B_, 5-HT_1D_, 5-HT_5_, and 5-HT_7_ receptors in mammals [66] and in insects [11,67,68]. Additional serotonergic ligands (e.g., the agonist 5-MT (IC_50_ = 20.6 nM/836 nM) and the antagonist mianserin (IC_50_ = 29.5 nM/21.5 nM)) were also potent blockers of the action of tyramine and octopamine on AmOctα2R. Mianserin is known for some time as a potent antagonist at octopamine receptors [69,70] and, more recently, was found to be an antagonist of the AmTAR2 receptor of the honeybee [30] and the PeaTAR1B receptor of the American cockroach, *P. americana* [71].

Overall, our results support the notion that octopamine signaling in insects is highly complex. It is noteworthy that octopamine receptors characterized so far have been shown to preferentially couple to G_s_ proteins to activate adenylyl cyclases and to G_q_-proteins, which induce intracellular Ca^2+^ mobilization (for reviews, see [2,4,72]. However, α_2_-adrenergic-like octopamine receptors have been found to inhibit adenylyl cyclase activity ([33,34] and this study), a property reminiscent of the phylogenetically related mammalian α_2_-adrenergic receptors (for a review, see [64]) and insect type 1 tyramine receptors (e.g., [27,28,47,71,73,74,75]). Whether the signaling properties of a given receptor in a cell line illustrates its typical behavior in a natural background would require experimental testing in native cell or tissue samples. To the best of our knowledge, only stimulatory actions of octopamine on adenylyl cyclase activity have been reported so far for native tissues of the honeybee [27,72] and other insects [76,77,78,79,80] as well as for insect cells lines [81,82,83]. We speculate that the inhibitory effects of α_2_-adrenergic-like octopamine receptors on adenylyl cyclase activity are masked by the effects of the more prominent α_1_-adrenergic-like and β-adrenergic-like octopamine receptors. To test this hypothesis, native cells, tissue, or organs should be identified that express AmOctα2R as the only phenolamine receptor. An alternative could be the use of well-characterized pharmacological tools that permit the selective and efficient activation or inhibition of one or the other receptor in preparations expressing more than one phenolamine receptor. We have successfully used such a strategy to disentangle the signaling pathways of 5-HT_2_ and 5-HT_7_ serotonin receptors, which are co-expressed in blowfly (*Calliphora vicina*) salivary glands [84]. In any case, the characterization of the signaling properties of a sixth member of the octopamine receptor family presented here for the honeybee should facilitate future in vivo pharmacological studies coupled with behavioral testing in this eusocial model organism.

## 4. Materials and Methods

### 4.1. Amplification of the Honeybee α2-Adrenergic-Like Octopamine Receptor (AmOctα2R) cDNA and Construction of pcAmOctα2R-HA Expression Vector

Total RNA was extracted from 10 brains of honeybee foragers using the RNeasy Plus Micro Kit (Qiagen, Hilden, Germany). Synthesis of cDNA was carried out with M-MLV Reverse Transcriptase (Invitrogen/ThermoFisher Scientific, Dreieich, Germany). For the amplification of the entire coding region of *AmOctα2R*, specific primers were designed based on available sequence information ([35]; GenBank accession number XM_001122075): sense primer 5′-CGAGGAATTCCACCATGCCGCTCCTCGGCACC-3′; antisense primer 5′-GACGTCTAGATTATGCATAGTCGGGGACGTCATAGGGATATTTGAAGAGTATCCTGCGG-3′ (eurofins, Ebersberg, Germany). Primers were designed to enable ligation into pcDNA3.1(+) vector (Invitrogen/ThermoFisher Scientific, Dreieich, Germany) and heterologous expression of AmOctα2R in eukaryotic cells. In the sense primer, an EcoRI restriction site and a Kozak consensus motif (CCACC; [85]) were inserted in front of the translational start codon. In the antisense primer, the receptor-encoding sequence was extended in a frame with a sequence encoding the hemagglutinin A (HA) tag to allow monitoring of receptor protein expression using specific anti-HA antibodies (Roche/Sigma-Aldrich/Merck, Darmstadt, Germany). In addition, an XbaI recognition sequence was introduced immediately after the stop codon (TAA). PCR was performed using the following protocol: 95 °C for 10 min, 35 cycles at 95 °C for 30 s, 65 °C for 30 s, and 72 °C for 150 s and a final extension at 72 °C for 5 min. The PCR product was separated by agarose gel electrophoresis. The fragment was excised from the gel, cleaned using a PCR clean-up and gel extraction kit (Macherey-Nagel, Düren, Germany), double-restricted with EcoRI and XbaI and cloned into the pcDNA3.1(+) vector. The expression construct (*pcAmOctα2R-HA*) was verified by sequencing on both strands (eurofins).

### 4.2. Multiple Sequence Alignment and Phylogenetic Analysis

For phylogenetic analysis, we included amino acid sequences of biogenic amine receptors of various protostomian and deuterostomian species. Sequences were obtained from NCBI databases (NCBI, Bethesda, MD, USA). Multiple amino acid sequence alignment was consequently trimmed to regions encoding TM 1–4, TM 5, TM 6, and TM 7 using ClustalW. Afterwards, evolutionary analyses were conducted in MEGA7 [86]. The evolutionary history was inferred using the neighbor-joining method [87] with 10,000-fold bootstrap resampling. The human rhodopsin sequence formed the outgroup.

The sequence identity and similarity of α_2_-adrenergic-like octopamine receptors between *A. mellifera, B. mori*, and *D. melanogaster* were determined by using BioEdit v. 7.0.5.3 [88] after pairwise alignment.

### 4.3. Functional Expression of the AmOctα2R-HA Receptor

For AmOctα2R-HA expression and pharmacological analysis, we used a human embryonic kidney (HEK293; flpIn cells; Invitrogen/ThermoFisher Scientific; #750-07)-based cell line that was transfected with a gene encoding a variant of the A2-subunit of the olfactory CNG ion channel [89] (flpTM cells), provided by Sibion biosciences, Jülich, Germany. These flpTM cells were transfected with 3 µg, 8 µg, or 12 µg of the *pcAmOctα2R-HA* construct by a modified calcium phosphate method [90] following a previously established protocol [48]. Transfected cells were selected in the presence of the antibiotics G418 (1 mg/mL) and hygromycin (100 µg/mL). Expression of AmOctα2R-HA was monitored by Western blotting and immunocytochemistry using anti-HA antibodies (Roche/Sigma-Aldrich/Merck).

### 4.4. Functional Analysis of the AmOctα2R-HA Receptor

A stably transfected cell line was used to examine AmOctα2R-HA receptor activity by Ca^2+^ imaging. Control measurements were performed in the parental (flpTM) cell line. Changes in [cAMP]_i_ were registered indirectly via co-expressed CNG channels that were opened by cAMP and cause an influx of extracellular Ca^2+^ [30,32,48]. Changes in [Ca^2+^]_i_ were monitored with the Ca^2+^-sensitive fluorescent dye Fluo-4. Cells were grown in 96-well dishes to a density of approximately 2 × 10^4^ cells per well and were loaded at room temperature with Fluo-4 AM as described previously [32]. After 90 min, the loading solution was substituted with a dye-free extracellular solution (ECS; 120 mM NaCl, 5 mM KCl, 2 mM MgCl_2_, 2 mM CaCl_2_, 10 mM HEPES, and 10 mM glucose, pH 7.4 (NaOH)) containing 100 µM IBMX. The plate was transferred into a fluorescence reader (FLUOstar Omega, BMG Labtech, Ortenberg, Germany) to monitor Fluo-4 fluorescence. The excitation wavelength was 485 nm. Fluorescence emission was detected at 520 nm. Concentration series of various biogenic amines and synthetic receptor ligands were added, once Fluo-4 fluorescence intensity reached a stable value in each well. The changes in Fluo-4 fluorescence were recorded automatically. Concentration–response curves were established from at least three independent experiments with quadruplicate measurements. Data were analyzed and displayed using Prism 5.04 software (GraphPad, San Diego, CA, USA).

### 4.5. Western Blot analysis

Membrane proteins from AmOctα2R-HA-expressing cells and non-transfected flpTM cells were prepared as described previously [32]. Briefly, cells were lysed in buffer A (10 mM NaCl, 25 mM HEPES (pH 7.5), 2 mM EDTA, and a mammalian protease inhibitor cocktail diluted at 1:500 (mPIC; Sigma-Aldrich/Merck, Darmstadt, Germany)). After centrifugation, membrane proteins were solubilized from the pellet with buffer B (100 mM NaCl, 25 mM HEPES pH 7.5, mPIC protease inhibitor (dilution, 1:500) and 1% (*w*/*v*) (3-((3-cholamidopropyl)-dimethylammonio)-1-propanesulfonate, (CHAPS)). Proteins (30 µg per lane) were separated by sodium dodecyl sulfate-polyacrylamide gel electrophoresis (SDS-PAGE; 10% gel) and transferred onto a polyvinylidene fluoride membrane (PVDF, Merck/Millipore, Darmstadt, Germany). Nonspecific binding sites were blocked by incubation for 30 min in 5% (*w*/*v*) dry milk in phosphate buffered saline (PBS; 130 mM NaCl, 7 mM Na_2_HPO_4_, and 3 mM NaH_2_PO_4_; pH: 7.4). The membrane was incubated with primary antibodies (anti-HA; dilution, 1:1000; Roche/Sigma-Aldrich/Merck) in PBS containing 0.02% (*v*/*v*) Tween-20 (PBT) overnight at 4 °C. After rinsing the membrane three times with PBT for 15 min each, secondary antibodies conjugated to horseradish peroxidase (donkey anti-rat-HRP; dilution, 1:5000 (Sigma-Aldrich/Merck, Darmstadt, Germany)) in PBT containing 0.5% (*w*/*v*) dry milk were added for 1 h at room temperature. After rinsing the membrane three times with PBT for 15 min each and two times with PBS for 5 min each, signals were visualized with an enhanced chemiluminescence detection system (Western Bright™-Kit; Advansta; San Jose, CA, USA) on Hyperfilm™ ECL (GE Healthcare/Merck, Darmstadt, Germany).

## Figures and Tables

**Table 1:** Mean values for the half-maximal stimulation (EC_50_ [M] and logEC_50_ ± SD) for octopamine and tyramine on AmOctα2R-HA. Values were obtained from the nonlinear fitting of the data (*n* = the number of experiments) from concentration–response curves (GraphPad Prism 5.04).

	Octopamine (*n* = 7)	Tyramine (*n* = 11)
EC_50_ [M]	5.87 × 10^−8^	1.85 × 10^−6^
logEC_50_	−7.43 ± 0.24	−5.78 ± 0.17

**Table 2:** Mean values for the half-maximal inhibition (IC_50_ [M] and logIC_50_ ± SD) for substances with antagonistic activity on tyramine-activated AmOctα2R. Values were obtained from the nonlinear fitting of the data (*n* = number of experiments) from concentration–response curves (GraphPad Prism 5.04).

Substance	Specificity in Humans ^1^	IC_50_ [M]	logIC_50_	Maximal Inhibition [%]	*n*
5-CT	agonist at 5-HT_1A_, 5-HT_1B_, 5-HT_1D_, 5-HT_5_, and 5-HT_7_ receptors	4.16 × 10^−9^	−8.48 ± 0.20	10.8	3
phentolamine	nonselective α-adrenergic antagonist	5.63 × 10^−^^9^	−8.30 ± 0.20	16.1	3
epinastine	nonsedating histamine H_1_ receptor antagonist	1.98 × 10^−^^8^	−7.75 ± 0.29	17.31	6
5-MT	agonist at 5-HT_1_, 5-HT_2_, 5-HT_4_, 5-HT_6_, and 5-HT_7_ receptors	2.06 × 10^−^^8^	−7.72 ± 0.20	20.3	4
mianserin	antagonist at the histamine H_1_, 5-HT_1D_, 5-HT_2A_, 5-HT_2C_, 5-HT_3_, 5-HT_6_, 5-HT_7_, α_1_-adrenergic and α_2_-adrenergic receptors	2.95 × 10^−^^8^	−7.71 ± 0.31	21.5	5
yohimbine	high affinity for the α_2_-adrenergic receptor, moderate affinity for the α_1_-adrenergic, 5-HT_1A_, 5-HT_1B_, 5-HT_1D_, 5-HT_1F_, 5-HT_2B_, and D_2_ receptors, and weak affinity for the 5-HT_1E_, 5-HT_2A_, 5-HT_5A_, 5-HT_7_, and D_3_ receptors; behaves as an antagonist at α_1_-adrenergic, α_2_-adrenergic, 5-HT_1B_, 5-HT_1D_, 5-HT_2A_, 5-HT_2B_ and D_2_, and as a partial agonist at 5-HT_1A_	8.14 × 10^−^^8^	−7.12 ± 0.32	14.3	4
ketanserin	affinity for multiple GPCR; antagonist at 5-HT_2A_ and 5-HT_2C_ receptors; high affinity for α_1_-adrenergic receptors and very high affinity for histamine H_1_ receptors; moderate affinity for α_2_-adrenergic and 5-HT_6_ receptors as well as weak affinity for dopamine D_1_ and D_2_ receptors	5.14 × 10^−^^7^	−6.29 ± 0.29	17.11	3
8-OH-DPAT	standard selective 5-HT_1A_ agonist; also has moderate affinity for 5-HT_7_ receptors	1.09 × 10^−^^6^	−6.15 ± 0.36	7.21	3
AS-19	agonist at the 5-HT_7_ receptor	no effect			6

^1^ These data have been obtained from the websites of Tocris (https://www.tocris.com/) and/or Sigma-Aldrich (https://www.sigmaaldrich.com).

## Supplementary Materials

Supplementary materials can be found at https://www.mdpi.com/1422-0067/21/24/9334/s1.

## Abbreviations

5-CT	5-carboxamidotryptamine
5-MT	5-methoxytryptamine
5-HT	5-hydroxytryptamine, serotonin
8-OH-DPAT	8-Hydroxy-2-(dipropylamino)tetralin
GPCR	G protein-coupled receptor
HA	hemagglutinin A
IBMX	3-isobutyl-1-methylxanthine
NKH477	water-soluble forskolin analog
RFU	relative fluorescence unit
TM	transmembrane
