# Supplementary material for: AmOctα2R: Functional Characterization of a Honeybee Octopamine Receptor Inhibiting Adenylyl Cyclase Activity
Source: Int J Mol Sci. 2020 Dec 8;21(24):9334. doi: 10.3390/ijms21249334 (PMC7762591; doi:10.3390/ijms21249334)
Supplement: Supplementary file 1 [file ijms-21-09334-s001.pdf]

Article

AmOctα2R: functional characterization of a honeybee octopamine receptor inhibiting adenylyl cyclase activity

Wolfgang Blenau, Joana Alessandra Wilms, Sabine Balfanz and Arnd Baumann

Supplementary Material

```
AmOctα2R     ATGCCGCTCCTCGGCACCATTATGACCATCGACGCCGCCGGTAGTCCGACTACGGCGTCC 60

AmOctα2R     AACAGCGTCCTCGACGAGAACATGACGTGGCCTCGACCGACCGGTGGCTATACGTCTTCC 120
DmOctα2R-L   ---------------------ATGGATTATAGCCGACTGAAT------------------ 21
DmOctα2R-S   ---------------------ATGGATTATAGCCGACTGAAT------------------ 21

AmOctα2R     GCTAACGAGCCACACGCGATCGACACCGTCACCATCCTCACCGACGACCGCCACCGACCA 180
DmOctα2R-L   ------------GCCAACATCAATACGGGCAACATCAGCACCGATGACTTTCTGGCCGTC 69
DmOctα2R-S   ------------GCCAACATCAATACGGGCAACATCAGCACCGATGACTTTCTGGCCGTC 69

AmOctα2R     CTGTCCACGACCGGACCCGACCACGTGGCCACGACACTCGTCGATCGTGCTAGCACCGGC 240
DmOctα2R-L   TTCTCGACCGGAAAGCCGGACAAT------------------------------------ 93
DmOctα2R-S   TTCTCGACCGGAAAGCCGGACAAT------------------------------------ 93

AmOctα2R     CCAACACCCAGCGTCCCGGCCGATCGGCGACCACCGGCCACCGACCAGGAGACCGAGATC 300
DmOctα2R-L   ------------------GCCACCCTCAATCCGCCCCTGCTCAGTGTCGATGGTCAGCTG 135
DmOctα2R-S   ------------------GCCACCCTCAATCCGCCCCTGCTCAGTGTCGATGGTCAGCTG 135

AmOctα2R     GCCGCTCTTCCACCGACCACCACCGAGGAGATCGAGACTGAAACCGAAACCGAGATCGAG 360
DmOctα2R-L   ACCCTGCCGCCGGGATCG------------------------------------------ 153
DmOctα2R-S   ACCCTGCCGCCGGGATCG------------------------------------------ 153

AmOctα2R     ATCGAGAGGAGCACGACATCGGGAGAAGAACAACGAGCGGAAGAAGAGGAGGATTGTGCC 420
DmOctα2R-L   ------------------------------------------------------------ 153
DmOctα2R-S   ------------------------------------------------------------ 153

AmOctα2R     TGCGGCGGATACGAGGATACGAGCAGACTTCCCTTCCTCGTGGCCGGCGAGGATCTCGAG 480
DmOctα2R-L   ------GGATATGTCAATGTCAACGATACGATATTCTTCCTGAAT--------------- 192
DmOctα2R-S   ------GGATATGTCAATGTCAACGATACGATATTCTTCCTGAAT--------------- 192

AmOctα2R     GCTGGAAGCAACAACACTGGGAGCGTGGACGGTAGCAGCCCGGGAAGTATCATCGAGGAA 540
DmOctα2R-L   ------------------GGCAGCTTCTACAACAGCAGC---------------CTGCAG 219
DmOctα2R-S   ------------------GGCAGCTTCTACAACAGCAGC---------------CTGCAG 219

AmOctα2R     GCTAGCACGGGGGTCGGTGGTACGAGCACGACGAGCACGAGCACGAGCACGGATCTGGAA 600
DmOctα2R-L   CTGGCGGCGGGATTCTATAATCAAAGCAGTGCTTCCGGCGCGACATCGGGCAATTTGACC 279
DmOctα2R-S   CTGGCGGCGGGATTCTATAATCAAAGCAGTGCTTCCGGCGCGACATCGGGCAATTTGACC 279

AmOctα2R     GACGTGGAAGGATTAAGGGAGTTGGAAGTGCTGTTCGGGTTGAACGGCATAATGGAGGAC 660
DmOctα2R-L   AATCCGAAT--------------------------------------------------- 288
DmOctα2R-S   AATCCGAAT--------------------------------------------------- 288
```

```
AmOctα2R     CTGGATGGATCGTTCAACGACTCTTTCAACGAGAGTCTATTGTTCCCGTGCAACGGTAGC 720
DmOctα2R-L   ------------------------------------------------------------ 288
DmOctα2R-S   ------------------------------------------------------------ 288

AmOctα2R     CTGTGCAACGAGACGTACGTGGTGCTCGGGAGAGCCTGTACCCGTCCGGGTACACGATT 780
DmOctα2R-L   ------------CACACAGAAGTCCACTGGGATGGCCGGTATCCCAGTGGATATACGCTT 336
DmOctα2R-S   ------------CACACAGAAGTCCACTGGGATGGCCGGTATCCCAGTGGATATACGCTT 336

AmOctα2R     CTTCAAATAGTCCTGGCGTCCGTCCTCGTCACGCTGTTGATGATCGTGATCGTGGTCGGG 840
DmOctα2R-L   ACGCACATCGTGATCGCCTCCATTATTGTGACGATCCTGATGATTATCATCGTGGTGGCC 396
DmOctα2R-S   ACGCACATCGTGATCGCCTCCATTATTGTGACGATCCTGATGATTATCATCGTGGTGGCC 396

AmOctα2R     AACATGCTTGTGATAATCGCGATCGCGACCGAGAAGGCGCTGAAAAACATACAGAATTGG 900
DmOctα2R-L   AACATGCTGGTGATCATAGCCATTGCCACGGAGAAGTCGTTGAAGAATATACAGAACTGG 456
DmOctα2R-S   AACATGCTGGTGATCATAGCCATTGCCACGGAGAAGTCGTTGAAGAATATACAGAACTGG 456

AmOctα2R     TTCATAGCGAGTTTGGCGGTGGCCGATTTCTTCCTAGGCCTCGTAATCATGCCCTTCTCG 960
DmOctα2R-L   TTCATTGCCTCGTTGGCGGTGGCCGACTTCTTTCTGGGCCTCATCATCATGCCATTTTCG 516
DmOctα2R-S   TTCATTGCCTCGTTGGCGGTGGCCGACTTCTTTCTGGGCCTCATCATCATGCCATTTTCG 516

AmOctα2R     TTGGCCAACGAGATCATGGGATATTGGATCTTTGGGTACTGGTGGTGCGACATTTACTCG 1020
DmOctα2R-L   CTGGCCAATGAGCTAATGGGCTACTGGATCTTCGGCAGCTGGTGGTGTGACATCCACTCC 576
DmOctα2R-S   CTGGCCAATGAGCTAATGGGCTACTGGATCTTCGGCAGCTGGTGGTGTGACATCCACTCC 576

AmOctα2R     GCCATGGACGTGTTGCTGTGCACGGCGAGCATCATGAACTTGTGTCTGATAAGCTTGGAC 1080
DmOctα2R-L   GCGATGGACGTCCTCCTCTGCACCGCCTCGATCATGAACCTGTGCCTCATCTCGTTAGAT 636
DmOctα2R-S   GCGATGGACGTCCTCCTCTGCACCGCCTCGATCATGAACCTGTGCCTCATCTCGTTAGAT 636

AmOctα2R     AGGTACTGGAGCATCACGCAGGCGGTGGATTATCTGAAGAAGAGAACTCCCGCAAGAGCG 1140
DmOctα2R-L   CGCTACTGGAGCATCACCAAGGCCGTCGACTATCTGAAGTCGAGAACGCCGGCGCGAGCC 696
DmOctα2R-S   CGCTACTGGAGCATCACCAAGGCCGTCGACTATCTGAAGTCGAGAACGCCGGCGCGAGCC 696

AmOctα2R     GCCCTCATGATCGCGCTTGTGTGGCTCCTGTCCGCCCTCGTTTGCATCCCCCCCCTCCTC 1200
DmOctα2R-L   GCCGTCATGATCACGGCAGTCTGGATTATGTCCGCCCTCATCTGCATACCGCCGCTCCTC 756
DmOctα2R-S   GCCGTCATGATCACGGCAGTCTGGATTATGTCCGCCCTCATCTGCATACCGCCGCTCCTC 756

AmOctα2R     GGATGGAAGAGACCCACGCCGGCTGAAGAGTATCCCAAGTGTAAG-Intron 1-CTTTC 1250
DmOctα2R-L   GGCTGGAAGGTGAAGATGCCAGAGGACCGCTGCCCAAGTGCGAG-Intron 1-CTAAG 806
DmOctα2R-S   GGCTGGAAGGTGAAGATGCCAGAGGACCGCTGCCCAAGTGCGAG-Intron 1-CTAAG 806

AmOctα2R     AGAGGACATAGGCTACGTCCTTTACTCGGCGCTCGGCAGCTTCTACATCCCGTCGTGCAT 1310
DmOctα2R-L   CGAGGACATTGGCTACGTGCTGTACTCGGCACTGGGCTCCTTCTACATACCCAGTTGCAT 866
DmOctα2R-S   CGAGGACATTGGCTACGTGCTGTACTCGGCACTGGGCTCCTTCTACATACCCAGTTGCAT 866

AmOctα2R     CATGGTGTTCGTGTACATACGCATTTACTTCGCAGCGAAGGCGCGGGCGCGGCGCGGGAT 1370
DmOctα2R-L   CATGGTCTTTGTGTACATACGAATTTACTTTGCCGCCAAGGCGCGGGCGAGGAGAGGCAT 926
DmOctα2R-S   CATGGTCTTTGTGTACATACGAATTTACTTTGCCGCCAAGGCGCGGGCGAGGAGAGGCAT 926

AmOctα2R     CCGGAAGCCGCGTCCACGCGCGGTCGTGCCGCCC----------GAGTCGCCGGACGTCA 1420
DmOctα2R-L   TAAAAAGCATCCCCGCAAAACGAACAACGAACAG-Intron 2-GTAACGAGCTTCACCA 976
DmOctα2R-S   TAAAAAGCATCCCCGCAAAACGAACAACGAACAG-Intron 2-GTAACGAGCTTCACCA 976

AmOctα2R     GGCAGACGAGCTTCACGCAGAGCACGCCCGCCACCGAGGCGAAGAAGCCGCCTTCCGGCT 1480
DmOctα2R-L   CCGCCAAGAAAGGAACAATACCGATGCCCTCCTCGAGCGGCGTGTCCGCTTTGCAGCTGC 1036
DmOctα2R-S   CCGCCAAGAAAGGAACAATACCGATGCCCTCCTCGAGCGGCGTGTCCGCTTTGCAGCTGC 1036
```

```
AmOctα2R     CCGCCATGGAGAACGTCGCCACCATCGAGAACCAGCCC-------------------GTTC 1522
DmOctα2R-L   ACCAGCAGCGCCAAATAGCCACCATCGAGACACCACCGAACAGCGCCAGCCTACCGATGC 1096
DmOctα2R-S   ACCAGCAGCGCCAAATAGCCACCATCGAGACACCACCGAACAGCGCCAGCCTACCGATGC 1096

AmOctα2R     AGATACCCATCGTCACGTGCGACTTTGCCAGCGACGTCAGCACCTCCGAGGCG------- 1575
DmOctα2R-L   AGATACCCACGGTGACCATGGACCTGGCCTCCGACATATCCACCTCGGAGGCGGGGGAAC 1156
DmOctα2R-S   AGATACCCACGGTGACCATGGACCTGGCCTCCGACATATCCACCTCGGAGGCGGGGGAAC 1156

AmOctα2R     ------------------------------------------------------------ 1575
DmOctα2R-L   TGGAGGCGGTGGCCGCCCAAACGGTGCTTGCCTATGCAAATCCCAATGGTGCGGGCACGA 1216
DmOctα2R-S   TGGAGGCGGTGGCCGCCCAAACGGTGCTTGCCTATGCAAATCCCAATGGTGCGGGCACGA 1216

AmOctα2R     --------------------------------GATCCCGGAGGGGGGAGCAGTATCCCCA 1603
DmOctα2R-L   ATTCGGTCACCGTGGTGAGCAGCGGGAACGGGGGCCCTGGGGCGTCGGGTGTTCCACCCA 1276
DmOctα2R-S   ATTCGGTCACCGTGGTGAGCAGCGGGAACGGGGGCCCTGGGGCGTCGGGTGTTCCACCCA 1276

AmOctα2R     TGGAGGAGAAGGACACGCTCAAG-Intron 2-GTGACG--------------------- 1632
DmOctα2R-L   GCTCGCCGAAGGAGACGCTTCAG----------GTGAGCACGATAGCCACCGGTCGGGTG 1326
DmOctα2R-S   GCTCGCCGAAGGAGACGCTTCAG----------GTGAGCACGATAGCCACCGGTCGGGTG 1326

AmOctα2R     ---------ATGCCGGTTCCGGTGCAAAAGAGCAGC------------------------ 1659
DmOctα2R-L   GGTCTCAATGTGCCCGTGCCACCGTCCATGGGCAGCAATAGTTCCATCAATGTGCTGAAC 1386
DmOctα2R-S   GGTCTCAATGTGCCCGTGCCACCGTCCATGGGCAGCAATAGTTCCATCAATGTGCTGAAC 1386

AmOctα2R     ---------------------CTGAAGGCGACGCTGTCCGTGAACGGTGACGGGCAGCAA 1698
DmOctα2R-L   AACAACAACGGTACCGTGGCGATGGATGGAGGTGCCACCGCCGCCGGCGGGGGAAATGGT 1446
DmOctα2R-S   AACAACAACGGTACCGTGGCGATGGATGGAGGTGCCACCGCCGCCGGCGGGGGAAATGGT 1446

AmOctα2R     TCGAGCGTCCCGTCC--------------------------------------------- 1713
DmOctα2R-L   AGTGCTGTAGCGACGCCTGGTGGACTGCAGCAAACGGGCGGTAATGAGCTGCGCGTCTCT 1506
DmOctα2R-S   AGTGCTGTAGCGACGCCTGGTGGACTGCAGCAAACGGGCGGTAATGAGCTGCGCGTCTCT 1506

AmOctα2R     ------------CGATGCAGGGCGCCCAGCGTCGGTATCGACGTGGACATGGTGTCCGAG 1761
DmOctα2R-L   CCGATCGCCGGACGGTGTCGAGCCCTATCCGTGGGCGTGGACACGGACATGGTGAGTGAA 1566
DmOctα2R-S   CCGATCGCCGGACGGTGTCGAGCCCTATCCGTGGGCGTGGACACGGACATG--------- 1557

AmOctα2R     TTCGATCCGTCGTCGTCGGACAGCGGCGTCGTTTCGAGATGCGCGGTCGTCAAGCCGCTC 1821
DmOctα2R-L   TTCGATCCATCCAGCAGCGATTCGGCGTGGTCAGTCGCTGTGCCGTGGTCAAGCCGCTC 1626
DmOctα2R-S   -------------------------truncation in exon 3--------------- 1557

AmOctα2R     AAGCTTCGCCTCTGCCAGCCCATATTCGGGCGGAGGAACATCGGCAAGCTGAGAAGGGAG 1881
DmOctα2R-L   AAGTTTCGCCTTTGCCAGCCGATCTTCGGGCGCAAGTCGAGTAACCAGCAGCGGCGCAAC 1686
DmOctα2R-S   ------------------CCGATCTTCGGGCGCAAGTCGAGTAACCAGCAGCGGCGCAAC 1599

AmOctα2R     CACGGGGGGACGGGGCCGC------------------GCGGGCGGCAAGGACGAGGCG 1923
DmOctα2R-L   GAGGCCAAAGCGGCGGCCAAGAATCAACAGTCCAGTGGAGTGGCCAGCAAAAATCAGCAG 1746
DmOctα2R-S   GAGGCCAAAGCGGCGGCCAAGAATCAACAGTCCAGTGGAGTGGCCAGCAAAAATCAGCAG 1659

AmOctα2R     GGC-----------------------------------------------GGCGAGGCC 1935
DmOctα2R-L   CAGCAGCAGCAGCAACAGCAGCAGCAGGTAGTAAAGTCGGAACTGGAGCCGGCCATACCG 1806
DmOctα2R-S   CAGCAGCAGCAGCAACAGCAGCAGCAGGTAGTAAAGTCGGAACTGGAGCCGGCCATACCG 1719

AmOctα2R     AAGTCGTCCAAGCCGCGGGACCCGGAGAGAGAGAAGAGGAGACTGGCGAGGAAGAAGGAG 1995
DmOctα2R-L   AAGACACCGAAGCCCAGGGATCCGGAGAAGGAGAAGCGACGAATTGCGCGAAAGAAGGAG 1866
DmOctα2R-S   AAGACACCGAAGCCCAGGGATCCGGAGAAGGAGAAGCGACGAATTGCGCGAAAGAAGGAG 1779
```

```
AmOctα2R     AAACGGGCCACGCTGATACTCGGCTTCATAATGGGCAGCTTCATAGCTTGCTGGCTGCCG 2055
DmOctα2R-L   AAGCGGGCCACCCTAATTCTGGGCCTGATCATGGGCAGCTTCATCGCCTGCTGGCTGCCC 1926
DmOctα2R-S   AAGCGGGCCACCCTAATTCTGGGCCTGATCATGGGCAGCTTCATCGCCTGCTGGCTGCCC 1839

AmOctα2R     TTCTTCGTCCTCTACATCGCGAAACCCCTCTTCCCG---AACGTCAAGATACCGATCCAG 2112
DmOctα2R-L   TTCTTCTTCCTGTACATCCTGGTGCCGGCATGTAGCAGCCATTGCAACATACCGGAATCG 1986
DmOctα2R-S   TTCTTCTTCCTGTACATCCTGGTGCCGGCATGTAGCAGCCATTGCAACATACCGGAATCG 1899

AmOctα2R     GCGTTCGTCATCGCTTTCTGGCTCGGTTACATGAACTCTGCCCTCAACCCCTTCATATAC 2172
DmOctα2R-L   GCGTTCGCCGTCGCCTTCTGGCTGGGCTACATGAATTCGGCGCTCAATCCGGCCATCTAT 2046
DmOctα2R-S   GCGTTCGCCGTCGCCTTCTGGCTGGGCTACATGAATTCGGCGCTCAATCCGGCCATCTAT 1959

AmOctα2R     ACCGTCTTCAACAAGGACTTCCGCAGGGCTTTCCGCAGGATACTCTTCAAATAA 2226
DmOctα2R-L   ACCATCTTCAACAAGGACTTCCGACGCGCCTTCCGGCGCATCCTGTTCAAGTGA 2100
DmOctα2R-S   ACCATCTTCAACAAGGACTTCCGACGCGCCTTCCGGCGCATCCTGTTCAAGTGA 2013
```

Figure S1. Nucleic acid sequence alignment of *AmOctα2R* and the two transcripts of *DmOctα2R* showing the position of introns. The genomic organization was analyzed using Splign [1]. The coding region of both *AmOctα2R* and the long variant of the *DmOctα2R* transcript (*DmOctα2R-L*) is interrupted by two introns. Whereas the position of intron 1 is conserved between both receptors, this is not the case for intron 2. The shorter variant of the *DmOctα2R* transcript (*DmOctα2R-S*) has a truncated exon 3, originating from the use of an alternative splice site [2]. For *AmOctα2R*, such a truncated transcript was not be detected in the honeybee brain. Whereas the consensus sequence for a splice donor site (**G**-**G**-[cut]-**G**-**T**-**R**-**A**-**G**-**T**) is found at the respective position in *DmOctα2R* (T-**G**-[cut]-**G**-**T**-**G**-**A**-**G**-**T**), this motif is not well conserved in *AmOctα2R* (T-**G**-[cut]-**G**-**T**-**G**-T-C-C).

Table S1. Genomic organization of the Am*Octα2R* gene. The table shows characteristics of the exons. Nucleotide sequences of introns are represented in lower-case letters and the nucleotide sequences of exons in upper case letters. The genomic organization was analyzed using Splign [1].

exon	length	cDNA position	genomic position	splice acceptor	splice donor
I	4.691 bp	1 - 4.691	501 - 5.192	-	GTAAG/gtaag
II	381 bp	4.692 - 5.072	104.678 - 105.058	tccag/CTTTC	TCAAG/gttga
III	6.631 bp	5.073 - 11.703	107.157 - 113.787	gacag/GTGAC	-

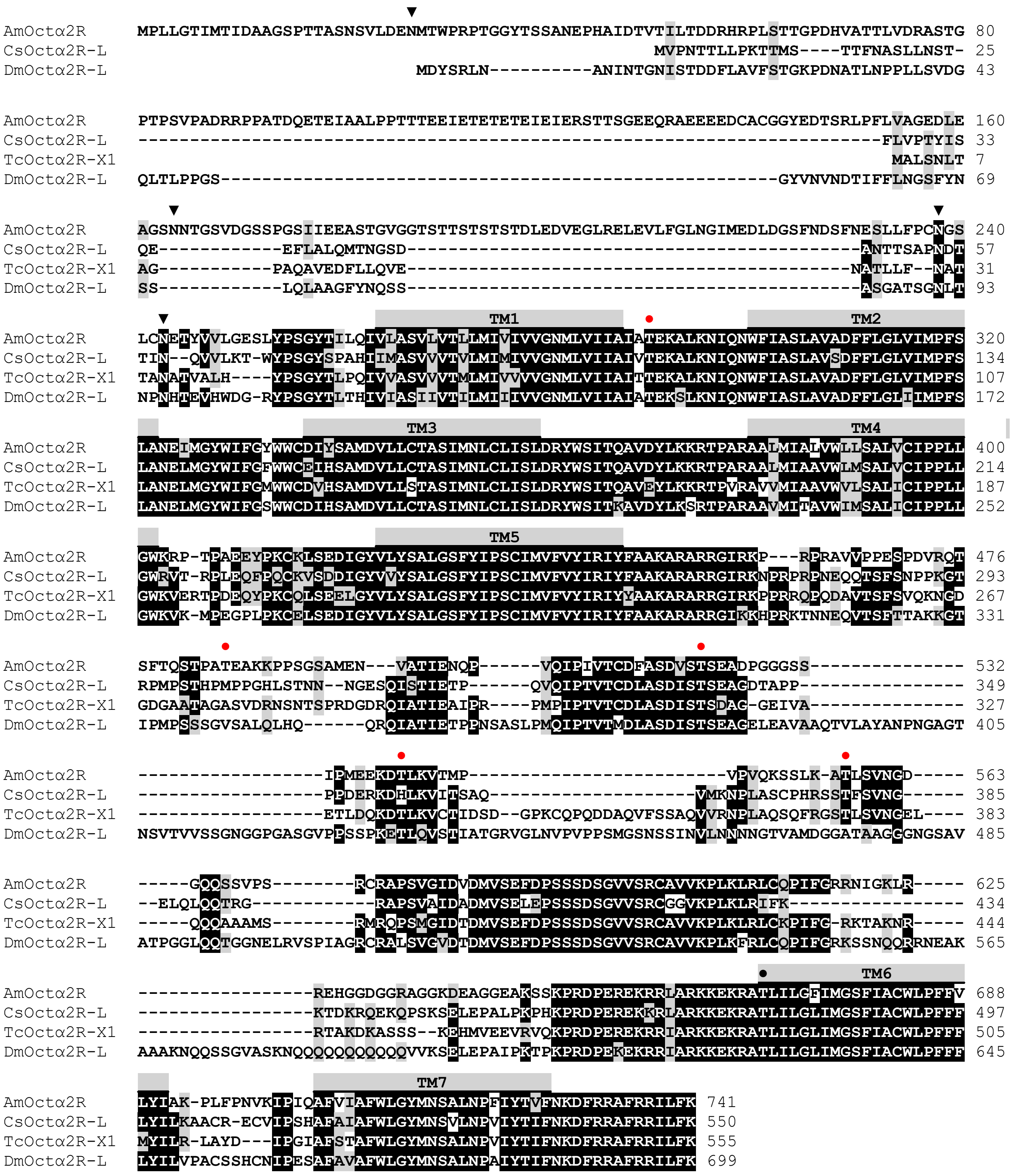

Figure S2. Amino acid sequence alignment of AmOctα2R and α_2-adrenergic-like octopamine receptor from *Chilo suppressalis* (CsOctα2R-L; AIC75370.1), *Tribolium castaneum* (TcOctα2R-L; XP_015839170.1), and *D. melanogaster* (DmOctα2R-L; CG18208; NP_650754.2). Identical residues (≥75%) are shown as white letters against black, whereas conservatively substituted residues are shaded. Grey bars indicate putative transmembrane domains (TM1-7). Potential posttranslational modification sites are labeled: *N*-glycosylation (black triangle), protein kinase C phosphorylation (red dots), protein kinase A phosphorylation (black dot). N-glycosylation sites were predicted by NetNGlyc 1.0 Server

(http://www.cbs.dtu.dk/services/NetNGlyc/). Putative phosphorylation sites were predicted by NetPhos 3.1 Server (http://www.cbs.dtu.dk/services/NetPhos/ [3]. The amino acid position is given on the right.

Table S2. Accession numbers and annotations of sequences used in the phylogenetic analysis.

ID	Accession No	Species	Description	Reference
**PROTOSTOMIA**, **Lophotrochozoa**, Annelida				
Pdα1	APC23842.1	*P. dumerilii*	α_1-adrenergic receptor	[4]
Pdα2	APC23843.1	*P. dumerilii*	α_2-adrenergic receptor	[4]
PdTAR1	AKQ63052.1	*P. dumerilii*	tyramine receptor 1	[4]
PdTAR2	APC23184.1	*P. dumerilii*	tyramine receptor 2	[4]
PdOctα1R	APC23183.1	*P. dumerilii*	α_1-adrenergic-like octopamine receptor	[4]
PdOctβR	APC23841.1	*P. dumerilii*	β-adrenergic-like octopamine receptor	[4]
**PROTOSTOMIA**, **Ecdysozoa**, Priapulida				
Pcα1	XP_014662992.1	*P. caudatus*	α_{1A}-adrenergic receptor-like	
Pcα2	XP_014681069.1	*P. caudatus*	α_{2C}-adrenergic receptor-like	
**PROTOSTOMIA**, **Ecdysozoa**, Arthropoda				
PaTAR1A	CAQ48240.1	*P. americana*	tyramine receptor 1A	[5]
PaTAR1B	SNT95699.1	*P. americana*	tyramine receptor 1B	[6]
PaOctα1R	AAP93817.1	*P. americana*	α_1-adrenergic-like octopamine receptor	[7]
PaDOP2A	CDK37789.1	*P. americana*	invertebrate-type dopamine receptor, isoform A	[8]
PaDOP3	AFH53996.1	*P. americana*	D_2-like dopamine receptor	
Pa5-HT1	CAX65666.1	*P. americana*	5-HT_1-like serotonin receptor	[9]
AmTAR1	NP_001011594.1	*A. mellifera*	tyramine receptor 1	[10]
AmTAR2	APL96716.1	*A. mellifera*	tyramine receptor 2	[11]
AmOctα1R	NP_001011565.1	*A. mellifera*	α_1-adrenergic-like octopamine receptor	[12]
AmOctα2R	XP_001122075.3	*A. mellifera*	α_2-adrenergic-like octopamine receptor	This work
AmOctβ1R	CCO13922.1	*A. mellifera*	β-adrenergic-like octopamine receptor 1	[13]
AmOctβ2R	CCO13923.1	*A. mellifera*	β-adrenergic-like octopamine receptor 2	[13]
AmOctβ3R	CCO13924.1	*A. mellifera*	β-adrenergic-like octopamine receptor 3	[13]
AmOctβ4R	CCO13925.1	*A. mellifera*	β-adrenergic-like octopamine receptor 4	[13]
AmDOP1	NP_001011595.1	*A. mellifera*	D_1-like dopamine receptor	[14]
AmDOP2	NP_001011567.1	*A. mellifera*	invertebrate-type dopamine receptor	[15,16]
AmDOP3	NP_001014983.1	*A. mellifera*	D_2-like dopamine receptor	[17]
Am5-HT1A	CBI75449.1	*A. mellifera*	5-HT_1-like serotonin receptor	[18]
Am5-HT2α	CBX90120.1	*A. mellifera*	5-HT_2-like serotonin receptor	[19]
Am5-HT2B	CBX90121.1	*A. mellifera*	5-HT_2-like serotonin receptor	[19]
Am5-HT7	CAJ28210.1	*A. mellifera*	5-HT_7-like serotonin receptor	[20]

DmTAR1	NP_524419.2	*D. melanogaster*	tyramine receptor 1 (octopamine-tyramine receptor)	[21]
DmTAR2	NP_650652.1	*D. melanogaster*	tyramine receptor 2	[22]
DmOctα1AR	CAB38026.1	*D. melanogaster*	α_1-adrenergic-like octopamine receptor 1, splice variant 1A	[23]
DmOctα1BR	CAB38025.1	*D. melanogaster*	α_1-adrenergic-like octopamine receptor 1, splice variant 1B	[23]
DmOctα2R	NP_650754.2	*D. melanogaster*	α_2-adrenergic-like octopamine receptor	[2]
DmOctβ1R	NP_651057.1	*D. melanogaster*	β-adrenergic-like octopamine receptor 1	[23,24]
DmOctβ2R	NP_001034049.1	*D. melanogaster*	β-adrenergic-like octopamine receptor 2	[24]
DmOctβ3R	NP_001034043.2	*D. melanogaster*	β-adrenergic-like octopamine receptor 2	[24]
DmDOP1	CAA54451.1	*D. melanogaster*	D_1-like dopamine receptor	[25]
DmDOP2	NP_733299.1	*D. melanogaster*	invertebrate-type dopamine receptor	[26]
DmDOP3	AAX52464.2	*D. melanogaster*	D_2-like dopamine receptor	[27]
Dm5-HT1A	CAA77570.1	*D. melanogaster*	5-HT_1-like serotonin receptor	[28]
Dm5-HT1B	CAA77571.1	*D. melanogaster*	5-HT_1-like serotonin receptor	[28]
Dm5-HT2α	NP_524223.2	*D. melanogaster*	5-HT_2-like serotonin receptor	[29]
Dm5-HT2β	NP_001262373.1	*D. melanogaster*	5-HT_2-like serotonin receptor	[30]
Dm5-HT7	NP_524599.1	*D. melanogaster*	5-HT_7-like serotonin receptor	[31]
**DEUTEROSTOMIA**, **Ambulacraria**, Priapulida				
Skα1	ALR88680.1	*S. kowalevskii*	α_1-adrenergic receptor-like 067	[32]
Skα2	XP_002734932.1	*S. kowalevskii*	α_{2C}-adrenergic receptor-like	
SkTAR1	XP_002742354.2	*S. kowalevskii*	tyramine receptor 1	
SkTAR2A	XP_002734062.1	*S. kowalevskii*	tyramine receptor 2A	
SkTAR2A	XP_006812999.1	*S. kowalevskii*	tyramine receptor 2B	
SkOctα1R	XP_006823182.1	*S. kowalevskii*	α_1-adrenergic-like octopamine receptor	
SkOctβR	XP_002733926.1	*S. kowalevskii*	β-adrenergic-like octopamine receptor	
**DEUTEROSTOMIA**, **Chordata**, Vertebrata				
Hsα1A	NP_000671.2	*H. sapiens*	α_{1A}-adrenergic receptor	[33]
Hsα1B	NP_000670.1	*H. sapiens*	α_{1B}-adrenergic receptor	[34]
Hsα1D	NP_000669.1	*H. sapiens*	α_{1D}-adrenergic receptor	[35]
Hsα2A	NP_000672.3	*H. sapiens*	α_{2A}-adrenergic receptor	[36]
Hsα2B	NP_000673.2	*H. sapiens*	α_{2B}-adrenergic receptor	[37]
Hsα2C	NP_000674.2	*H. sapiens*	α_{2C}-adrenergic receptor	[38]
Hsβ1	NP_000675.1	*H. sapiens*	β_1-adrenergic receptor	[39]
Hsβ2	NP_000015.1	*H. sapiens*	β_2-adrenergic receptor	[40]
Hsβ3	NP_000016.1	*H. sapiens*	β_3-adrenergic receptor	[41]
HsD1A	NP_000785.1	*H. sapiens*	D_{1A} dopamine receptor	[42]
HsD1B	NP_000789.1	*H. sapiens*	D_{1B} dopamine receptor	[43]
HsD2	NP_000786.1	*H. sapiens*	D_2 dopamine receptor isoform long	[44]
HsD3	NP_000787.2	*H. sapiens*	D_3 dopamine receptor isoform a	[45]
HsD4	NP_000788.2	*H. sapiens*	D_4 dopamine receptor	[46]
Hs5-HT1A	NP_000515.2	*H. sapiens*	5-HT_{1A} serotonin receptor	[47]
Hs5-HT1B	NP_000854.1	*H. sapiens*	5-HT_{1B} serotonin receptor	[48]

Hs5-HT1D	NP_000855.1	*H. sapiens*	5-HT_{1D} serotonin receptor	[49]
Hs5-HT1E	NP_000856.1	*H. sapiens*	5-HT_{1E} serotonin receptor	[50]
Hs5-HT1F	NP_000857.1	*H. sapiens*	5-HT_{1F} serotonin receptor	[51]
Hs5-HT2A	NP_000612.1	*H. sapiens*	5-HT_{2A} serotonin receptor isoform 1	[52]
Hs5-HT2B	NP_000858.3	*H. sapiens*	5-HT_{2B} serotonin receptor isoform 1	[53]
Hs5-HT2C	NP_000859.1	*H. sapiens*	5-HT_{2C} serotonin receptor 2C isoform a precursor	[52]
Hs5-HT7	NP_000863.1	*H. sapiens*	5-HT_{7} serotonin receptor isoform a	[54]
HsRHOD	AAC31763.1	*H. sapiens*	rhodopsin	[55]

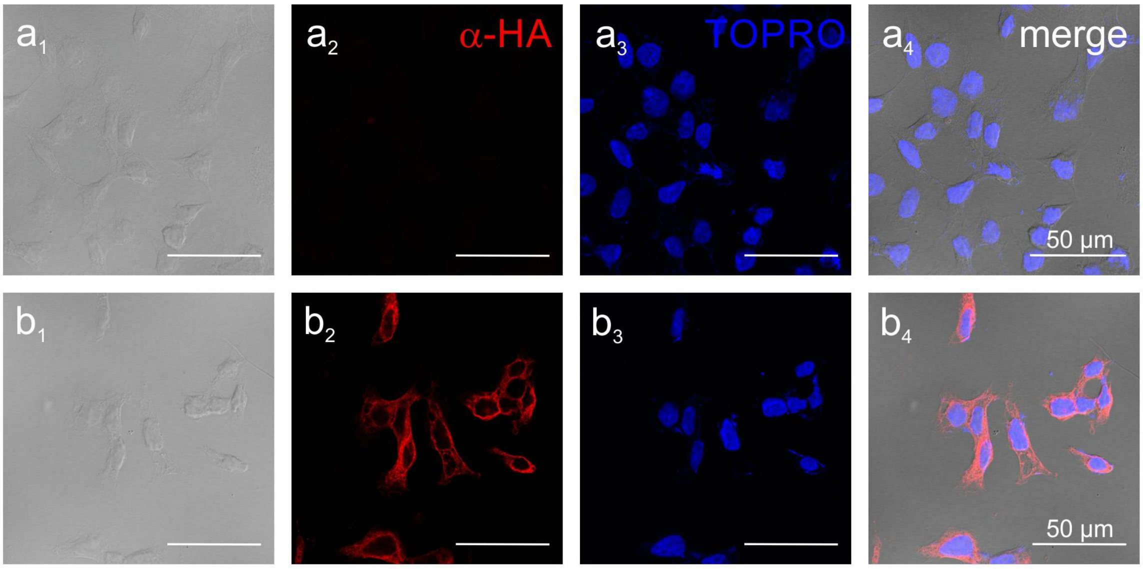

Figure S3. Confocal microscopy of AmOctα2R-expressing flpTM cells. Non-transfected (a) and AmOctα2R-expressing (b) cells were stained with rat anti-HA antibodies. (a_1+b_1) DIC microscopy. (a_2+b_2) Cells were fixed with 4% (v/v) paraformaldehyde, rinsed with PBS buffer and blocked with 5% (v/v) ChemiBLOCKER (Merck, Darmstadt, Germany) and 0.5% (v/v) Triton X-100 in PBS. Samples were incubated with primary rat anti-HA antibodies (dilution 1:100) and secondary donkey anti-rat-Cy3 (dilution 1:400) antibodies. Non-transfected flpTM cells do not express the AmOctα2R protein. In AmOctα2R-expressing flpTM cells (b_2), the AmOctα2R protein could be detected in the plasma membrane and in the cytosol. (a_3+b_3): Nuclei were stained with the dye TO-PRO and are clearly differentiated from the cytosol. (a_4+b_4): Composite images.

Table S3. Mean values for half-maximal inhibition (IC50 [M] and log IC50 ± SD) for substances with antagonistic activity on octopamine-activated AmOctα2R. For each substance at least two independent experiments with quadruplicate measurements were performed. Values were obtained from non-linear fitting of the data from concentration-response curves (GraphPad Prism 5.04).

Substance	IC_{50} [M]	log IC_{50}
5-CT	2.69×10^{-10}	-9.57 ± 0.21
phentolamine	8.21×10^{-9}	-8.09 ± 0.13
epinastine	1.61×10^{-8}	-7.79 ± 0.19
5-MT	8.36×10^{-7}	-6.08 ± 0.37
mianserin	2.15×10^{-8}	-7.67 ± 0.25
yohimbine	3.65×10^{-7}	-6.44 ± 0.09
ketanserin	3.25×10^{-7}	-6.49 ± 0.32
8-OH-DPAT	1.66×10^{-7}	-6.78 ± 0.45

References

1. Kapustin, Y.; Souvorov, A.; Tatusova, T.; Lipman, D. Splign: algorithms for computing spliced alignments with identification of paralogs. *Biol Direct.* **2008**, *3*, 20.
2. Qi, Y.X.; Xu, G.; Gu, G.X.; Mao, F.; Ye, G.Y.; Liu, W.; Huang, J. A new *Drosophila* octopamine receptor responds to serotonin. *Insect Biochem. Mol. Biol.* **2017**, *90*, 61-70.
3. Blom, N.; Sicheritz-Pontén, T.; Gupt,a R.; Gammeltoft, S.; Brunak, S. Prediction of post-translational glycosylation and phosphorylation of proteins from the amino acid sequence. *Proteomics* **2004**, 4, 1633-1649.
4. Bauknecht, P.; Jékely, G. Ancient coexistence of norepinephrine, tyramine, and octopamine signaling in bilaterians. *BMC Biol.* **2017**, *15*, 6.
5. Rotte, C.; Krach, C.; Balfanz, S.; Baumann, A.; Walz, B.; Blenau, W. Molecular characterization and localization of the first tyramine receptor of the American cockroach (*Periplaneta americana*). *Neuroscience* **2009**, *162*, 1120-1133.
6. Blenau, W.; Balfanz, S.; Baumann, A. PeaTAR1B: Characterization of a second type 1 tyramine receptor of the American cockroach, *Periplaneta americana*. *Int. J. Mol. Sci.* **2017**, *18*, 2279.
7. Bischof, L.J.; Enan, E.E. Cloning, expression and functional analysis of an octopamine receptor from *Periplaneta americana*. *Insect Biochem. Mol. Biol.* **2004**, *34*, 511-521.
8. Troppmann, B.; Balfanz, S.; Krach, C.; Baumann, A.; Blenau, W. Characterization of an invertebrate-type dopamine receptor of the American cockroach, *Periplaneta americana*. *Int. J. Mol. Sci.* **2014**, *15*, 629-653.
9. Troppmann, B.; Balfanz, S.; Baumann, A.; Blenau, W. Inverse agonist and neutral antagonist actions of synthetic compounds at an insect 5-HT_1 receptor. *Br. J. Pharmacol.* **2010**, *159*, 1450-1562.
10. Blenau, W.; Balfanz, S.; Baumann, A. Amtyr1: characterization of a gene from honeybee (*Apis mellifera*) brain encoding a functional tyramine receptor. *J. Neurochem.* **2000**, *74*, 900-908.
11. Reim, T.; Balfanz, S.; Baumann, A.; Blenau, W.; Thamm, M.; Scheiner, R. AmTAR2: Functional characterization of a honeybee tyramine receptor stimulating adenylyl cyclase activity. *Insect Biochem. Mol. Biol.* **2017**, *80*, 91-100.
12. Grohmann, L.; Blenau, W.; Erber, J.; Ebert, P.R.; Strünker, T.; Baumann, A. Molecular and functional characterization of an octopamine receptor from honeybee (*Apis mellifera*) brain. *J. Neurochem.* **2003**, *86*, 725-735.
13. Balfanz, S.; Jordan, N.; Langenstück, T.; Breuer, J.; Bergmeier, V.; Baumann, A. Molecular, pharmacological, and signaling properties of octopamine receptors from honeybee (*Apis mellifera*) brain. *J. Neurochem.* **2014**, *129*, 284-296.
14. Blenau, W.; Erber, J.; Baumann, A. Characterization of a dopamine D_1 receptor from *Apis mellifera*: cloning, functional expression, pharmacology, and mRNA localization in the brain. *J. Neurochem.* **1998**, *70*, 15-23.
15. Mustard, J.A.; Blenau, W.; Hamilton, I.S.; Ward, V.K.; Ebert, P.R.; Mercer, A.R. Analysis of two D1-like dopamine receptors from the honey bee *Apis mellifera* reveals agonist-independent activity. *Brain Res. Mol. Brain Res.* **2003**, *113*, 67-77.
16. Humphries, M.A.; Mustard, J.A.; Hunter, S.J.; Mercer, A.; Ward, V.; Ebert, P.R. Invertebrate D_2 type dopamine receptor exhibits age-based plasticity of expression in the mushroom bodies of the honeybee brain. *J. Neurobiol.* **2003**, *55*, 315-330.
17. Beggs, K.T.; Hamilton, I.S.; Kurshan, P.T.; Mustard, J.A.; Mercer, A.R. Characterization of a D2-like dopamine receptor (AmDOP3) in honey bee, *Apis mellifera*. *Insect Biochem. Mol. Biol.* **2005**, *35*, 873-882.

18. Thamm, M.; Balfanz, S.; Scheiner, R.; Baumann, A.; Blenau, W. Characterization of the 5-HT_{1A} receptor of the honeybee (*Apis mellifera*) and involvement of serotonin in phototactic behavior. *Cell. Mol. Life Sci.* **2010**, *67*, 2467-2479.
19. Thamm, M.; Rolke, D.; Jordan, N.; Balfanz, S.; Schiffer, C.; Baumann, A.; Blenau, W. Function and distribution of 5-HT_2 receptors in the honeybee (*Apis mellifera*). *PLoS One* **2013**, *8*, e82407.
20. Schlenstedt, J.; Balfanz, S.; Baumann, A.; Blenau, W. Am5-HT_7: molecular and pharmacological characterization of the first serotonin receptor of the honeybee (*Apis mellifera*). *J. Neurochem.* **2006**, *98*, 1985-1998.
21. Kutsukake, M.; Komatsu, A.; Yamamoto, D.; Ishiwa-Chigusa, S. A tyramine receptor gene mutation causes a defective olfactory behavior in *Drosophila melanogaster*. *Gene* **2000**, *245*, 31-42.
22. Cazzamali, G.; Klaerke, D.A.; Grimmelikhuijzen, C.J. A new family of insect tyramine receptors. *Biochem. Biophys. Res. Commun.* **2005**, *338*, 1189-1196.
23. Balfanz, S.; Strünker, T.; Frings, S.; Baumann, A. A family of octopamine receptors that specifically induce cyclic AMP production or Ca^{2+} release in *Drosophila melanogaster*. *J. Neurochem.* **2005**, *93*, 440-451.
24. Maqueira, B.; Chatwin, H.; Evans, P.D. Identification and characterization of a novel family of *Drosophila* β-adrenergic-like octopamine G-protein coupled receptors. *J. Neurochem.* **2005**, *94*, 547-560.
25. Gotzes, F.; Balfanz, S.; Baumann, A. Primary structure and functional characterization of a *Drosophila* dopamine receptor with high homology to human $D_{1/5}$ receptors. *Receptors Channels* **1994**, *2*, 131-141.
26. Feng, G.; Hannan, F.; Reale, V.; Hon, Y.Y.; Kousky, C.T.; Evans, P.D.; Hall, L.M. Cloning and functional characterization of a novel dopamine receptor from *Drosophila melanogaster*. *J. Neurosci.* **1996**, *16*, 3925-3933.
27. Hearn, M.G.; Ren, Y.; McBride, E.W.; Reveillaud, I.; Beinborn, M.; Kopin, A.S. A *Drosophila* dopamine 2-like receptor: Molecular characterization and identification of multiple alternatively spliced variants. *Proc. Natl. Acad. Sci. U. S. A.* **2002**, *99*, 14554-14559.
28. Saudou, F.; Boschert, U.; Amlaiky, N.; Plassat, J.L.; Hen, R. A family of *Drosophila* serotonin receptors with distinct intracellular signalling properties and expression patterns. *EMBO J.* **1992**, *11*, 7-17.
29. Colas, J.F.; Launay, J.M.; Kellermann, O.; Rosay, P.; Maroteaux, L. *Drosophila* 5-HT_2 serotonin receptor: coexpression with fushi-tarazu during segmentation. *Proc. Natl. Acad. Sci. U. S. A.* **1995**, *92*, 5441-5445.
30. Blenau, W.; Stöppler, D.; Balfanz, S.; Thamm, M.; Baumann, A. Dm5-HT_{2B}: pharmacological characterization of the fifth serotonin receptor subtype of *Drosophila melanogaster*. *Front. Syst. Neurosci.* **2017**, *11*, 28.
31. Witz, P.; Amlaiky, N.; Plassat, J.L.; Maroteaux, L.; Borrelli, E.; Hen, R. Cloning and characterization of a *Drosophila* serotonin receptor that activates adenylate cyclase. *Proc. Natl. Acad. Sci. U. S. A.* **1990**, *87*, 8940-8944.
32. Simakov, O.; Kawashima, T.; Marlétaz, F.; Jenkins, J.; Koyanagi, R.; Mitros, T.; Hisata, K.; Bredeson, J.; Shoguchi, E.; Gyoja, F.; Yue, J.X.; Chen, Y.C.; Freeman, R.M. Jr.; Sasaki, A.; Hikosaka-Katayama, T.; Sato, A.; Fujie, M.; Baughman, K.W.; Levine, J.; Gonzalez, P.; Cameron, C.; Fritzenwanker, J.H.; Pani, A.M.; Goto, H.; Kanda, M.; Arakaki, N.; Yamasaki, S.; Qu, J.; Cree, A.; Ding, Y.; Dinh, H.H.; Dugan, S.; Holder, M.; Jhangiani, S.N.; Kovar, C.L.; Lee, S.L.; Lewis, L.R.; Morton, D.; Nazareth, L.V.; Okwuonu, G.; Santibanez, J., Chen, R., Richards, S., Muzny, D.M., Gillis, A.; Peshkin, L.; Wu, M.; Humphreys, T.; Su, Y.H.; Putnam, N.H.; Schmutz, J.; Fujiyama, A.; Yu, J.K.; Tagawa, K.; Worley, K.C.; Gibbs, R.A.; Kirschner, M.W.; Lowe, C.J.; Satoh, N.; Rokhsar, D.S.; Gerhart, J. Hemichordate genomes and deuterostome origins. *Nature* **2015**, *527*, 459-465.
33. Schwinn, D.A.; Lomasney, J.W.; Lorenz, W.; Szklut, P.J.; Fremeau, R.T. Jr.; Yang-Feng, T.L.; Caron, M.G.; Lefkowitz, R.J.; Cotecchia, S. Molecular cloning and expression of the cDNA for a novel α_1-adrenergic receptor subtype. *J. Biol. Chem.* **1990**, *265*, 8183-8189.
34. Allen, L.F.; Lefkowitz, R.J.; Caron, M.G.; Cotecchia, S. G-protein-coupled receptor genes as protooncogenes: constitutively activating mutation of the α_{1B}-adrenergic receptor enhances mitogenesis and tumorigenicity. *Proc. Natl. Acad. Sci. U. S. A.* **1991**, *88*, 11354-11358.
35. Bruno, J.F.; Whittaker, J.; Song, J.F.; Berelowitz. M. Molecular cloning and sequencing of a cDNA encoding a human α_{1A} adrenergic receptor. *Biochem. Biophys. Res. Commun.* **1991**, *179*, 1485-1490.
36. Kobilka, B.K.; Matsui, H.; Kobilka, T.S.; Yang-Feng, T.L.; Francke, U.; Caron, M.G.; Lefkowitz, R.J.; Regan, J.W. Cloning, sequencing, and expression of the gene coding for the human platelet α_2-adrenergic receptor. *Science* **1987**, *238*, 650-656.
37. Lomasney, J.W.; Lorenz, W.; Allen, L.F.; King, K.; Regan, J.W.; Yang-Feng, T.L.; Caron, M.G.; Lefkowitz, R.J. Expansion of the α_2-adrenergic receptor family: cloning and characterization of a human α_2-adrenergic receptor subtype, the gene for which is located on chromosome 2. *Proc. Natl. Acad. Sci. U. S. A.* **1990**, *87*, 5094-5098.
38. Regan, J.W.; Kobilka, T.S.; Yang-Feng, T.L.; Caron, M.G.; Lefkowitz, R.J.; Kobilka, B.K. Cloning and expression of a human kidney cDNA for an α_2-adrenergic receptor subtype. *Proc. Natl. Acad. Sci. U. S. A.* **1988**, *85*, 6301-6305.
39. Frielle, T.; Collins, S.; Daniel, K.W.; Caron, M.G.; Lefkowitz, R.J.; Kobilka, B. K. Cloning of the cDNA for the human β_1-adrenergic receptor. *Proc. Natl. Acad. Sci. U. S. A.* **1987**, *84*, 7920-7924.
40. Emorine, L.J.; Marullo, S.; Delavier-Klutchko, C.; Kaveri, S.V.; Durieu-Trautmann, O.; Strosberg, A.D. Structure of the gene for human β_2-adrenergic receptor: expression and promoter characterization. *Proc. Natl. Acad. Sci. U. S. A.* **1987**, *84*, 6995-6999.

41. Emorine, L.J.; Marullo, S.; Briend-Sutren, M.M.; Patey, G.; Tate, K.; Delavier-Klutchko, C.; Strosberg, A.D. Molecular characterization of the human β_3-adrenergic receptor. *Science* **1989**, *245*, 1118-1121.
42. Sunahara, R.K.; Niznik, H.B.; Weiner, D.M.; Stormann, T.M.; Brann, M.R.; Kennedy, J.L.; Gelernter, J.E.; Rozmahel, R.; Yang, Y.L.; Israel, Y.; Seeman, P.; O'Dowd, B.F. Human dopamine D_1 receptor encoded by an intronless gene on chromosome 5. *Nature* **1990**, *347*, 80-83.
43. Sunahara, R.K.; Guan, H.C.; O'Dowd, B.F.; Seeman, P.; Laurier, L.G.; Ng, G.; George, S.R.; Torchia, J.; Van Tol, H.H.; Niznik, H.B. Cloning of the gene for a human dopamine D_5 receptor with higher affinity for dopamine than D_1. *Nature* **1991**, *350*, 614-619.
44. Dearry, A.; Falardeau, P.; Shores, C.; Caron, M.G. D_2 dopamine receptors in the human retina: cloning of cDNA and localization of mRNA. *Cell. Mol. Neurobiol.* **1991**, *11*, 437-453.
45. Sokoloff, P.; Giros, B.; Martres, M.P.; Bouthenet, M.L.; Schwartz J.C. Molecular cloning and characterization of a novel dopamine receptor (D_3) as a target for neuroleptics. *Nature* **1990**, *347*, 146-151.
46. Van Tol, H.H.; Bunzow, J.R.; Guan, H.C.; Sunahara, R.K.; Seeman, P.; Niznik, H.B., Civelli, O. Cloning of the gene for a human dopamine D_4 receptor with high affinity for the antipsychotic clozapine. *Nature* **1991**, *350*, 610-614.
47. Fargin, A.; Raymond, J.R.; Lohse, M.J.; Kobilka, B.K.; Caron, M.G.; Lefkowitz, R.J. The genomic clone G-21 which resembles a β-adrenergic receptor sequence encodes the 5-HT_{1A} receptor. *Nature* **1988**, *335*, 358-360.
48. Jin, H.; Oksenberg, D.; Ashkenazi, A.; Peroutka, S.J.; Duncan, A.M.; Rozmahel, R.; Yang, Y.; Mengod, G.; Palacios, J.M.; O'Dowd, B.F. Characterization of the human 5-hydroxytryptamine$_{1B}$ receptor. *J. Biol. Chem.* **1992**, *267*, 5735-5738.
49. Hamblin, M.W.; Metcalf, M.A. Primary structure and functional characterization of a human 5-HT_{1D}-type serotonin receptor. *Mol. Pharmacol.* **1991**, *40*, 143-148.
50. Levy, F.O.; Gudermann, T.; Perez-Reyes, E.; Birnbaumer, M.; Kaumann, A.J.; Birnbaumer, L. Molecular cloning of a human serotonin receptor (S12) with a pharmacological profile resembling that of the 5-HT_{1D} subtype. *J. Biol. Chem.* **1992**, *267*, 7553-7562.
51. Amlaiky, N.; Ramboz, S.; Boschert, U.; Plassat, J.L.; Hen. R. Isolation of a mouse "5HT1E-like" serotonin receptor expressed predominantly in hippocampus. *J. Biol. Chem.* **1992**, *267*, 19761-19764.
52. Saltzman, A.G.; Morse, B.; Whitman, M.M.; Ivanshchenko, Y.; Jaye, M.; Felder, S. Cloning of the human serotonin 5-HT_2 and 5-HT_{1C} receptor subtypes. *Biochem. Biophys. Res. Commun.* **1991**, *181*, 1469-1478.
53. Schmuck, K.; Ullmer, C.; Engels, P.; Lubbert, H. Cloning and functional characterization of the human 5-HT_{2B} serotonin receptor. *FEBS Lett.* **1994**, *342*, 85-90.
54. Lovenberg, T.W.; Baron, B.M.; de Lecea, L.; Miller, J.D.; Prosser, R.A.; Rea, M.A.; Foye, P.E.; Racke, M.; Slone, A.L.; Siegel, B.W.; Danielson, P.A.; Sutcliffe, J.G.; Erlander M.G. A novel adenylyl cyclase-activating serotonin receptor (5-HT_7) implicated in the regulation of mammalian circadian rhythms. *Neuron* **1993**, *11*, 449-458.
55. Nathans, J.; Hogness, D.S. Isolation and nucleotide sequence of the gene encoding human rhodopsin. *Proc. Natl. Acad. Sci. U. S. A.* **1984**, *81*, 4851-4855.
